# Synergistic induction of phytoalexins in *Nicotiana attenuata* by jasmonate and ethylene signaling mediated by NaWRKY70

**DOI:** 10.1093/jxb/erad415

**Published:** 2023-10-23

**Authors:** Na Song, Jinsong Wu

**Affiliations:** Yunnan Key Laboratory for Wild Plant Resources, Kunming Institute of Botany, Chinese Academy of Sciences, Kunming, 650201, China; University of Chinese Academy of Science, Beijing 10049, China; Yunnan Key Laboratory for Fungal Diversity and Green Development, Kunming Institute of Botany, Chinese Academy of Sciences, Kunming, 650201, China; Yunnan Key Laboratory for Wild Plant Resources, Kunming Institute of Botany, Chinese Academy of Sciences, Kunming, 650201, China; Universitat Jaume I, Spain

**Keywords:** *Alternaria*, ethylene, jasmonate, *Nicotiana*, phytoalexin, scopoletin, scopolin, WRKY

## Abstract

Production of the phytoalexins scopoletin and scopolin is regulated by jasmonate (JA) and ethylene signaling in *Nicotiana* species in response to *Alternaria alternata*, the necrotrophic fungal pathogen that causes brown spot disease. However, how these two signaling pathways are coordinated to control this process remains unclear. In this study, we found that the levels of these two phytoalexins and transcripts of their key enzyme gene, *feruloyl-CoA 6ʹ-hydroxylase 1* (*NaF6ʹH1*), were synergistically induced in *Nicotiana attenuata* by co-treatment with methyl jasmonate (MeJA) and ethephon. By combination of RNA sequencing and virus-induced gene silencing, we identified a WRKY transcription factor, NaWRKY70, which had a similar expression pattern to *NaF6ʹH1* and was responsible for *A. alternata*-induced *NaF6ʹH1* expression. Further evidence from stable transformed plants with RNA interference, knock out and overexpression of *NaWRKY70* demonstrated that it is a key player in the synergistic induction of phytoalexins and plant resistance to *A. alternata*. Electrophoretic mobility shift, chromatin immunoprecipitation–quantitative PCR, and dual-luciferase assays revealed that NaWRKY70 can bind directly to the *NaF6ʹH1* promoter and activate its expression. Furthermore, the key regulator of the ethylene pathway, NaEIN3-like1, can directly bind to the *NaWRKY70* promoter and activate its expression. Meanwhile, NaMYC2s, important JA pathway transcription factors, also indirectly regulate the expression of *NaWRKY70* and *NaF6ʹH1* to control scopoletin and scopolin production. Our data reveal that these phytoalexins are synergistically induced by JA and ethylene signaling during *A. alternata* infection, which is largely mediated by NaWRKY70, thus providing new insights into the defense responses against *A. alternata* in *Nicotiana* species.

## Teaser

Levels of the phytoalexins scopoletin and scopolin in *Nicotiana* species are synergistically induced by jasmonate and ethylene signaling during *Alternaria alternata* infection, largely mediated by a WRKY transcription factor, NaWRKY70.

## Introduction

To adapt to a complex and dynamic environment, plants produce specialized secondary metabolites, of which there are hundreds of thousands different types. Many of these are responsible for growth, development, and plant immunity (Ahuja *et al.*, 2012; Vaughan *et al.*, 2015; Erb and Kliebenstein, 2020). Phytoalexins, a group of low molecular mass secondary metabolites produced after pathogen attack or elicitation, are important ‘chemical weapons’ in plant resistance to pathogens (Ahuja *et al.*, 2012; Wu, 2020). Camalexin is the most important phytoalexin for resistance to necrotrophic pathogens such as *Botrytis cinerea*, *Alternaria brassicicola*, and *Phytophthora brassicae* in Arabidopsis (Ferrari *et al.*, 2007; Nafisi *et al.*, 2007; Schlaeppi *et al.*, 2010). Capsidiol and scopoletin are two major phytoalexins produced by *Nicotiana* species in response to pathogens (El Oirdi *et al.*, 2010; Sun *et al.*, 2014b; Song *et al.*, 2019).

*Alternaria alternata* (tobacco pathotype), a necrotrophic fungal pathogen, is the causal agent of brown spot disease in *Nicotiana* species, including *Nicotiana tabacum* (LaMondia, 2001) and wild tobacco, *Nicotiana attenuata* (Schuck *et al.*, 2014; Sun *et al.*, 2014a). Previously, we have demonstrated that capsidiol, scopoletin, and scopolin are three conserved major phytoalexins produced in *Nicotiana* species in response to *A. alternata* (Sun *et al.*, 2014b; Li and Wu, 2016; Song *et al.*, 2019; Long *et al.*, 2021). Scopoletin, 7-hydroxy-6-methoxy-phenolic coumarin, is biosynthesized through the phenylpropanoid pathway (Kai *et al.*, 2006). Mutant and gene silencing analysis revealed that feruloyl-CoA 6ʹ-hydroxylase 1 (F6ʹH1) was the key enzyme for the formation of scopoletin and scopolin in Arabidopsis and *N. attenuata* (Kai *et al.*, 2008; Sun *et al.*, 2014b). Scopolin, a β-glycoside form of scopoletin, also acts as a phytoalexin against tobacco mosaic virus and *A. alternata* (Chong *et al.*, 2002; Li and Wu, 2016).

Jasmonate (JA) and ethylene are two important plant hormones involved in defense against necrotrophic fungi and herbivores (McDowell and Dangl, 2000; Onkokesung *et al.*, 2010). In response to pathogen or herbivore attack, plants synthesize JA–isoleucine (JA-Ile), the active form of jasmonate. JA-Ile can combine with the receptor complex SCF^COI1^, which ubiquitinates JASMONATE ZIM DOMAIN (JAZ) repressor proteins to release regulators, including MYC2, MYC3, and MYC4, thereby activating JA-regulated downstream response genes (Katsir *et al.*, 2008; Sheard *et al.*, 2010). Ethylene is perceived by a number of receptors that bind to constitutive ethylene response 1 (CTR1), a Raf-like kinase. The essential positive regulator ETHYLENE INSENSITIVE 2 (EIN2) acts downstream of CTR1. In the presence of ethylene, CTR1 is inactive, resulting in the accumulation of the transcription factors EIN3 and EIN3-like1 (EIL1), which subsequently activate various ethylene response genes (Yanagisawa *et al.*, 2003; Ju *et al.*, 2012). Previously, we showed that the production of scopoletin and scopolin was severely impaired in transgenic plants with silenced *NaAOC* or *NaACO*, two key enzyme genes for JA and ethylene biosynthesis (Sun *et al.*, 2014b; Li and Wu, 2016; Sun *et al.*, 2017), suggesting that both JA and ethylene signaling are crucial for the biosynthesis of scopoletin and scopolin. However, the detailed mechanism by which these two phytoalexins are coordinately regulated by JA and ethylene signaling remains unclear.

WRKY transcription factors, a large family of regulatory proteins only present in plants, are involved in growth, development, defense against pathogens, and response to external stimuli (Eulgem *et al.*, 2000; Chen *et al.*, 2012). They are characterized by the conserved WRKYGQK and zinc finger-like motifs that recognize W-box *cis*-elements in the promoter of target genes and activate or inhibit their expression (Rushton *et al.*, 2010; Chen *et al.*, 2012). In Arabidopsis, AtWRKY33 acts as a master transcription factor to directly regulate the expression of the camalexin biosynthesis genes *PAD3* and *CYP71A13* for defense against *B. cinerea* (Mao *et al.*, 2011), and CPK5/CPK6- and MPK3/MPK6-mediated differential phosphorylation of AtWRKY33 was found to be cooperatively involved in camalexin biosynthesis (Zhou *et al.*, 2020). Interestingly, analysis of the *NaF6ʹH1* promoter in *N. attenuata* revealed that its expression was likely regulated by some unknown WRKYs, as five W-boxes occurred in the promoter region. Recently, a NaWRKY3 was found to be a master regulator of JA, ethylene, and reactive oxygen species signaling in *N. attenuata* against *A. alternata* infection (Xu *et al.*, 2023). It would be very interesting to investigate whether any WRKYs can integrate JA and ethylene signaling to regulate scopoletin and scopolin biosynthesis.

Here, we showed that the levels of scopoletin and scopolin and the expression of their key enzyme gene *NaF6ʹH1* were remarkably and synergistically induced by JA and ethylene signaling in *N. attenuata*. We identified the NaWRKY70 transcription factor as a key integrator of JA and ethylene signaling to directly regulate *NaF6ʹH1* expression and scopoletin and scopolin biosynthesis. We also showed that NaMYC2s and NaEIN3-like1, two key regulators of the JA and ethylene pathways, both function as transcriptional regulators of *NaWRKY70* and activators of *NaF6ʹH1* expression and scopoletin and scopolin production. Our data provide new insights into the defense responses of *Nicotiana* plants after *A. alternata* attack.

## Materials and methods

### Plant and fungal materials

Seeds of the 35th generation of an *N. attenuata* inbred line were used as the wild-type (WT) genotype. The seeds of *N. attenuata* transgenic lines including irACO (ethylene reduced), Ov-etr1 (ethylene insensitive), irAOC (deficient in JA biosynthesis), and irCOI1 (JA-insensitive) plants were previously generated (Paschold *et al.*, 2007; von Dahl *et al.*, 2007; Kallenbach *et al.*, 2012), and provided by Prof. Ian T. Baldwin (Max-Planck Institute for Chemical Ecology). Seed germination and plant growth were conducted as described in Krügel *et al.* (2002). Briefly, seeds were sterilized in 2 ml dichloroisocyanuric acid (Sigma-Aldrich) for 5 min, rinsed with sterile water, followed by 2 ml liquid smoke (House of Herbs, Passaic, NY, USA; with the addition of 20 µl 0.1 M GA_3_) for 1 h. Treated seeds were germinated on GB_5_ medium for 10 d. The seedlings were transferred to 1 litre pots and grown to rosette-stage for experimental use in a greenhouse with 16 h light.

*Alternaria alternata* was grown on potato dextrose agar (PDA) medium and incubated at 28 °C for 5–7 d. The lamina of source–sink transition leaves (0-leaves) was inoculated with four PDA plugs on each leaf containing *A. alternata* for the indicated number of days. Leaf samples of around 1.5 × 1.5 cm^2^ with inoculation sites were harvested for the experiments (Sun *et al.*, 2014a).

### Hormone treatment

The 0-leaves of 32-day-old *N. attenuata* plants were sprayed with 1 mM methyl jasmonate (MeJA, Sigma-Aldrich) or 5 mM ethephon (2-chloroethanephosphonic acid, an ethylene-releasing agent; Sigma-Aldrich). Both MeJA and ethephon were prepared with distilled water. Leaves treated with H_2_O were used as negative controls. Samples were harvested at the indicated times for further analysis.

### RNA extract and quantitative PCR

Total RNA was isolated using TRI reagent (Thermo Fisher Scientific) and cDNA was synthesized as described in Wu *et al.* (2013). Quantitative PCR (qPCR) was performed on the CFX Connect qPCR instrument (Bio-Rad Laboratories) using iTaq Universal SYBR Green Supermix (Bio-Rad) and specific primers (Supplementary Table S1) according to the manufacturer’s instructions. A linear standard curve (obtained from threshold cycle number versus log cDNA amount) was obtained by using a series of dilutions of a specific cDNA sample, and the transcription levels of unknown samples were calculated according to this standard curve (Song *et al.*, 2019) and normalized with reference genes. We checked the three reference genes *Actin*, *Elongation factor 1-alpha*, and *60S ribosomal protein L23a* by qPCR in leaf samples treated with MeJA, ethephon or co-treatments of MeJA and ethephon for 1, 3, and 6 h, or inoculated with *A. alternata* for 1 or 3 d, or treated with *A. alternata* at different leaf positions for 1 d. The results showed that the *C*_t_ values of *Actin*, *Elongation factor 1-alpha*, and *60S ribosomal protein* were at the same level in all cDNA samples (reverse transcribed from 50 ng of total RNA) with different treatments (Supplementary Fig. S1). We therefore selected one of them, *Actin*, as the reference gene in this study.

### RNA-seq data analysis

After 6 h treatment with water control, MeJA (1 mM), ethephon (5 mM), and co-treatments of MeJA and ethephon, three biological replicates of WT source–sink transition leaves with the same treatments were mixed for RNA preparation. RNA sequencing was performed by Shanghai OE-Biotech (http://www.oebiotech.com/) using the Illumina Hiseq 2500. Sequencing was performed at 8 G depth and mapped to the *N. attenuata* reference genome sequence. Differential expression between each treatment with log2 (fold change)≥3 and its significance were calculated.

The raw sequence data reported in this paper have been deposited in the Genome Sequence Archive in BIG Data Center, Beijing Institute of Genomics (BIG), Chinese Academy of Sciences, under accession number CRA012626.

### Generation of virus-induced gene silencing plants

The highly specific fragments of the genes *NaMYC4-like*, *NaMYC2-like*, *NaMYB24-like*, *NaMYB57-like*, *NaHAT5-like*, *NabHLH18-like*, *NaERF17-like*, *NaMYB44-like*, *NaZinc655-like*, *NaWRKY70*, *NaMYB4-like*, *NaWRKY75-like*, *NaWRKY70-like*, *NaWRKY41-like*, *NabHLH137-like*, *NabHLH93-like*, *NaEIN3-like*, *NaEIN3-like1*, *NaMYC2a*, *NaMYC2b*, and *NaMYC2c* were amplified with specific primer pairs (Supplementary Table S1). The PCR fragments were digested with *Hin*dIII and *Bam*HI, and cloned into the pTV00 vector. *Agrobacterium tumefaciens* (strain GV3101) carrying each of these constructs was mixed with that with pBINTRA, and inoculated into *N. attenuata* leaves, generating target gene-silenced plants. *Agrobacterium tumefaciens*-mediated transformation was performed as previously described in Wu *et al.* (2008). The empty pTV00 vector (EV) and the pTV00 vector carrying *phytoene desaturase* were used as controls.

### Generation of the NaWRKY70-RNAi, -knockout, and -overexpression plants

The highly specific inverted sequence of *NaWRKY70* was amplified using primers (Supplementary Table S1) and inserted into the pRESC8 vector for RNA interference (RNAi). The full coding sequence of *NaWRKY70* was amplified using primers (Supplementary Table S1) and inserted into the pCAMBIA1301-eGFP vector for overexpression. The recombinant vectors were introduced into *N. attenuata* plants by transformation with *Agrobacterium tumefaciens* LBA4404. Single-insertion RNAi (NaWRKY70-RNAi-1^#^ and NaWRKY70-RNAi-4^#^) and overexpression lines (Ov-NaWRKY70-1^#^ and Ov-NaWRKY70-2^#^) were identified, bred to homozygosity in the T2 generation and used in this study. The NaWRKY70 knockout lines *NaWRKY70*-cas9-12^#^ and *NaWRKY70*-cas9-13^#^ were generated using the CRISPR/Cas9 system as described in Liang and Wu (2022).

### Subcellular localization of NaWRKY70

The coding sequence of *NaWRKY70* was cloned into the pM999 vector. *Nicotiana attenuata* protoplasts were prepared as described in Song *et al.* (2019). After incubation for 16–18 h, the signal of 35S::NaWRKY70-eGPF was observed using a fluorescence microscope (Leica DM5500 B). The empty vector pM999 was used as a negative control.

### Dual luciferase transcriptional activity assay

The promoters of *NaWRKY70* and *NaF6ʹH1* were amplified with specific primers and cloned into the pCAMBIA3301-LUC-REN reporter vector. The coding sequences of *NaEIN3-like1* and *NaWRKY70* were amplified with specific primers and inserted into the pCAMBIA3301 effector vector. The constructed reporter and effector vectors were transformed into *Agrobacterium tumefaciens* GV3101 strain, and transiently expressed in *N. benthamiana* leaves. Luciferase (LUC) and *Renilla* luciferase (REN) activities were measured using a dual luciferase assay kit (Yeasen Biotechnology). The LUC/REN ratio was used to determine the transcriptional activity of the promoter. Supplementary Table S1 lists the primers used. All experiments were performed with at least five biological replicates.

### Electrophoretic mobility shift assays

The full-length coding sequences of *NaWRKY70*, *NaEIN3-like*, *NaEIN3-like1*, *NaMYC2a*, *NaMYC2b*, and *NaMYC2c* were amplified with specific primers (Supplementary Table S1), cloned into the glutathione *S*-transferase (GST)-fusion pGEX-4T-1 vector and transformed into the *Escherichia coli* strain BL21. The fusion proteins were induced by isopropyl-β-d-thiogalactopyranoside (0.01–0.05 mM), and the bacterial cells were harvested and purified using GST-tag purification resin (Beyotime). Binding of recombinant protein and biotin-labeled probes was detected using a chemiluminescence electrophoretic mobility shift assay (EMSA) kit (Beyotime) according to the protocol suggested by the manufacturer.

### Quantification of scopoletin and scopolin

Leaf samples of approximately 0.2 g were harvested and ground to a fine powder in liquid nitrogen. The levels of scopoletin and scopolin were determined by HPLC–MS/MS as described in Sun *et al.* (2014b).

### Chromatin immunoprecipitation assays

Chromatin immunoprecipitation was performed using the EpiQuik Plant chromatin immunoprecipitation (ChIP) kit (Epigentek) according to the users’ manual. The source–sink transition leaves of *NaWRKY70-eGFP* overexpressing plants (1.5–2 g) infected with *A. alternata* were prepared for ChIP assays. Relative enrichments were measured by qPCR. All primers used in the ChIP experiments are listed in Supplementary Table S1. Chromatins were precipitated with IgG and with green fluorescent protein (GFP) antibodies. Primers detecting Actin served as the negative controls.

### Accession numbers

The GenBank accession numbers of the genes in this article are as follows: *NaMYC4-like* (XM_019377229.1), *NaMYC2-like* (*NaMYC2d*; XM_019380820.1), *NaMYB24-like* (XM_019373720.1), *NaMYB57-like* (XM_019370259.1), *NaHAT5-like* (XM_019367916.1), *NabHLH18-like* (XM_019373104.1), *NaERF17-like* (XM_019378163.1), *NaMYB44-like* (XM_019376996.1), *NaZinc finger 655-like* (XM_019390032.1), *NaWRKY70* (XM_019399160.1), *NaMYB4-like* (XM_019391424.1), *NaWRKY75-like* (XM_019404314.1), *NaWRKY70-like* (XM_019372971.1), *NaWRKY41-like* (XM_019374936.1), *NabHLH137-like* (XM_019380008.1), *NabHLH93-like* (XM_019401728.1), *NaEIN3-like* (XM_019382253.1), *NaEIN3-like1* (XM_019403482.1), *NaMYC2a* (XM_019398607.1), *NaMYC2b* (XM_019368208.1), *NaMYC2c* (XM_019375733.1), *NaCCoAOMT5* (XM_019376905.1), and *NaCCoAOMT6* (XM_019402596.1).

## Results

### Synergistic induction of phytoalexins scopoletin and scopolin by jasmonate and ethylene signaling

**Fig. 1.**
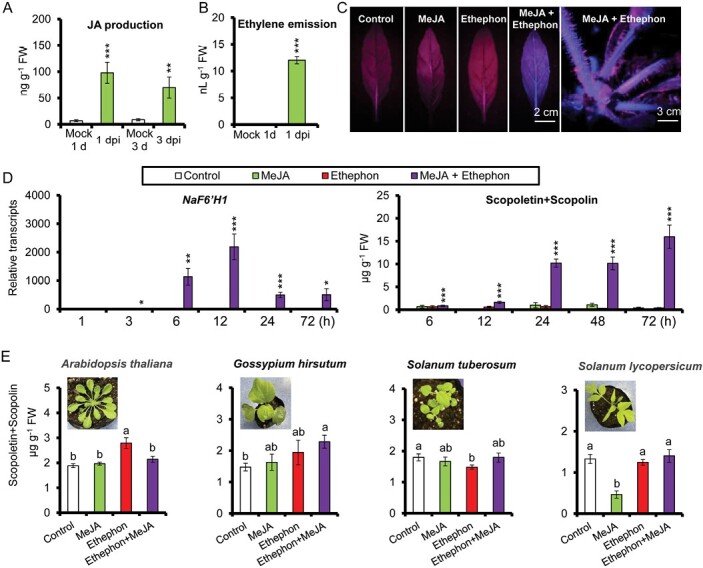
Synergistic induction of the phytoalexins scopoletin and scopolin by JA and ethylene signals in the genus of *Nicotiana.* (A) Mean (±SE) *A. alternata*-induced JA levels were measured by HPLC-MS/MS in six biological replicates of 0-leaves from WT plants at 1 and 3 days post-inoculation (dpi). Asterisks indicate the level of significant differences between mock and inoculated samples at 1 and 3 dpi (Student’s *t*-test: **P<0.01, ***P<0.005). (B) *Alternaria alternata*-induced ethylene production over 24 h was measured by GC-MS in four biological replicates of 0-leaves at 1 dpi. Asterisks indicate the level of significant differences between mock and inoculated samples (Student’s *t*-test: ***P<0.005). (C) Photographs of 0-leaves and whole WT plants under UV after 3 d treatment with water control, MeJA, ethephon, or co-treatment with MeJA and ethephon. The blue fluorescence of rosette 0-leaves (left panel) and whole plant (right panel) were evident under UV light when co-treated with MeJA and ethephon. (D) Left panel: mean (±SE) relative expression levels of *NaF6ʹH1* were measured in five biological replicates of 0-leaves at 1, 3, 6, 12, 24, and 72 h after treatments with water control, MeJA, ethephon, or co-treatment with MeJA and ethephon. Right panel: mean (±SE) scopoletin and scopolin levels were measured in five biological replicates of 0-leaves at 6, 12, 24, 48, and 72 h after treatments with water control, MeJA, ethephon, or co-treatment with MeJA and ethephon. Asterisks indicate the level of significant differences between control and samples co-treated with MeJA and ethephon at the same time points (Student’s *t*-test: *P<0.05, **P<0.01, ***P<0.005). (E) Mean (±SE) scopoletin and scopolin levels of five biological replicates were measured in Arabidopsis, *Gossypium hirsutum*, and the solanaceous plants *Solanum tuberosum* and *Solanum lycopersicum* when supplied with water control, MeJA, ethephon, or co-treatment of MeJA and ethephon. Different letters indicate significant differences by two-way ANOVA followed by Duncan’s test (*P*<0.05).

In response to *A. alternata*, *N. attenuata* plants activated both JA and ethylene signaling, both of which are required for the biosynthesis of the phytoalexins scopoletin and its β-glycoside form, scopolin (Fig. 1A, B) (Sun *et al.*, 2014b; Li and Wu, 2016; Sun *et al.*, 2017). Interestingly, these two phytoalexins could not be induced in leaves treated with either MeJA or ethephon alone. These results led us to hypothesize that scopoletin and scopolin could only be induced by activation of both JA and ethylene signaling. We therefore examined the accumulation of scopoletin and scopolin by exogenous co-treatment with MeJA and ethephon. Indeed, strong blue fluorescence, a signature of scopoletin and scopolin under UV light, was observed in the lamina of 0-leaves after co-treatment with MeJA and ethephon for 3 d, whereas no blue fluorescence was accumulated in leaves treated with MeJA or ethephon alone (Fig. 1C).

We next measured the transcripts of *NaF6ʹH1*, the key enzyme gene for scopoletin biosynthesis, by qPCR. Compared with water control treatments, *NaF6ʹH1* transcripts were strongly elicited up to 600-fold in 0-leaves treated simultaneously with MeJA and ethephon for 6 h, peaked at 2189-fold at 12 h, and gradually decreased to around 500-fold from 24 h to 72 h. However, *NaF6ʹH1* transcripts were not altered in 0-leaves when treated with MeJA or ethephon alone (Fig. 1D). Consistently, when leaves were co-treated with MeJA and ethephon simultaneously, scopoletin and scopolin levels increased significantly at 6 h, and peaked at 15.97 ± 2.53 µg g^−1^ fresh leaves at 72 h, whereas phytoalexin levels did not differ between leaves treated with water and MeJA or ethephon alone (Fig. 1D). In addition, co-treatment with MeJA and ethephon also increased the production of scopoletin and scopolin in the lamina of mature leaves and the midrib of young and mature leaves (Supplementary Fig. S2).

Interestingly, the synergistic induction of scopoletin and scopolin by MeJA and ethylene seemed to be conserved in the genus of *Nicotiana*. We also observed it in cultivated tobacco K326 (Wu and Song, 2021), but not in Arabidopsis or the solanaceous plants *Solanum tuberosum* and *Solanum lycopersicum* (Fig. 1E). In the case of *Gossypium hirsutum*, only a slight increase of blue fluorescence was observed in leaves after co-treatment with MeJA and ethephon (Fig. 1E).

Taken together, we observed the phenomenon of synergistic induction of *NaF6ʹH1* expression and scopoletin and scopolin production by MeJA and ethephon.

### Screening the key transcription factors involved in the synergistic induction of scopoletin and scopolin by jasmonate and ethylene signaling

To identify the key transcription factors involved in the synergistic induction of phytoalexins by JA and ethylene signaling, we performed a transcriptome analysis by RNA sequencing in 0-leaves of WT plants treated for 6 h with water control, MeJA, ethephon, or both MeJA and ethephon. Co-expression analysis revealed that 132 transcription factors (TFs) showed a synergistic induction of their expression by MeJA and ethephon co-treatments similar to *NaF6ʹH1*. Finally, 16 candidate TFs were enriched with log2 (fold change)≥3 compared with MeJA or ethephon alone (Supplementary Table 2).

**Fig. 2.**
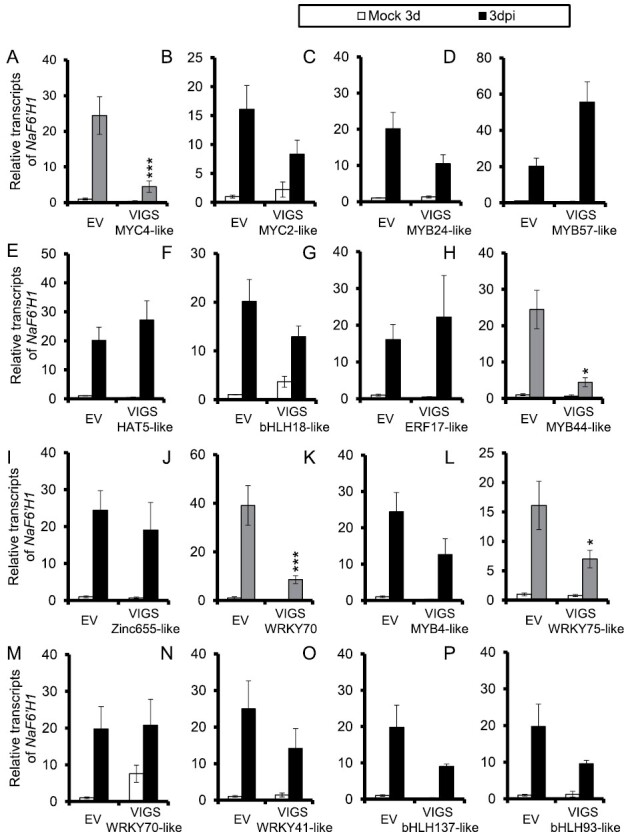
*Alternaria alternata*-elicited *NaF6ʹH1* expression measured in plants transformed with empty vector (EV) or silenced with 16 candidate TFs individually by VIGS. Mean (±SE) *A. alternata*-induced *NaF6ʹH1* transcripts were measured by qPCR in five biological replicates of young leaves of EV, VIGS NaMYC4-like (A), VIGS NaMYC2-like (B), VIGS NaMYB24-like (C), VIGS NaMYB57-like (D), VIGS NaHAT5-like (E), VIGS NabHLH18-like (F), VIGS NaERF17-like (G), VIGS NaMYB44-like (H), VIGS NaZinc655-like (I), VIGS NaWRKY70 (J), VIGS NaMYB4-like (K), VIGS NaWRKY75-like (L), VIGS NaWRKY70-like (M), VIGS NaWRKY41-like (N), VIGS NabHLH137-like (O), and VIGS NabHLH93-like (P) plants at 3 dpi. Asterisks indicate the level of significant difference between EV and VIGS plants after infection by *A. alternata* (Student’s *t*-test: *P<0.05, ***P<0.005).

We then silenced these 16 TFs individually by virus-induced gene silencing (VIGS) to identify those involved in scopoletin and scopolin biosynthesis, including *NaMYC4-like*, *NaMYC2-like*, *NaMYB24-like*, *NaMYB57-like*, *NaHAT5-like*, *NabHLH18-like*, *NaERF17-like*, *NaMYB44-like*, *NaZinc655-like*, *NaWRKY70*, *NaMYB4-like*, *NaWRKY75-like*, *NaWRKY70-like*, *NaWRKY41-like*, *NabHLH137-like*, and *NabHLH93-like*. Our results showed that *A. alternata*-elicited *NaF6ʹH1* expression was significantly reduced in plants silenced with *NaMYC4-like*, *NaMYB44-like*, *NaWRKY70*, or *NaWRKY75-like* (Fig. 2). Therefore, NaWRKY70 was selected as a candidate for further analysis in this study.

### *NaWRKY70* expression induced by *A. alternata* in a jasmonate/ethylene- and age-dependent manner

*NaWRKY70* encodes a peptide of 225 amino acids, containing a conserved WRKYGQK domain and a Cx_7_Cx_23_HxC zinc-finger (Supplementary Fig. S3A). When *NaWRKY70*::*eGFP*, which was driven by the CaMV 35s promoter, was transformed into *N. attenuata* protoplasts, strong GFP fluorescence was observed in the nucleus (Supplementary Fig. S3B). A phylogenetic tree of WRKYs from Arabidopsis, *Solanaceae*, and other plants, was constructed using the neighbor-joining (NJ) program. NaWRKY70, NtWRKY70, and NtWRKY4 were clustered together (Supplementary Fig. S3C). These results confirmed that NaWRKY70 is a member of the WRKY family, and is localized in the nucleus.

**Fig. 3.**
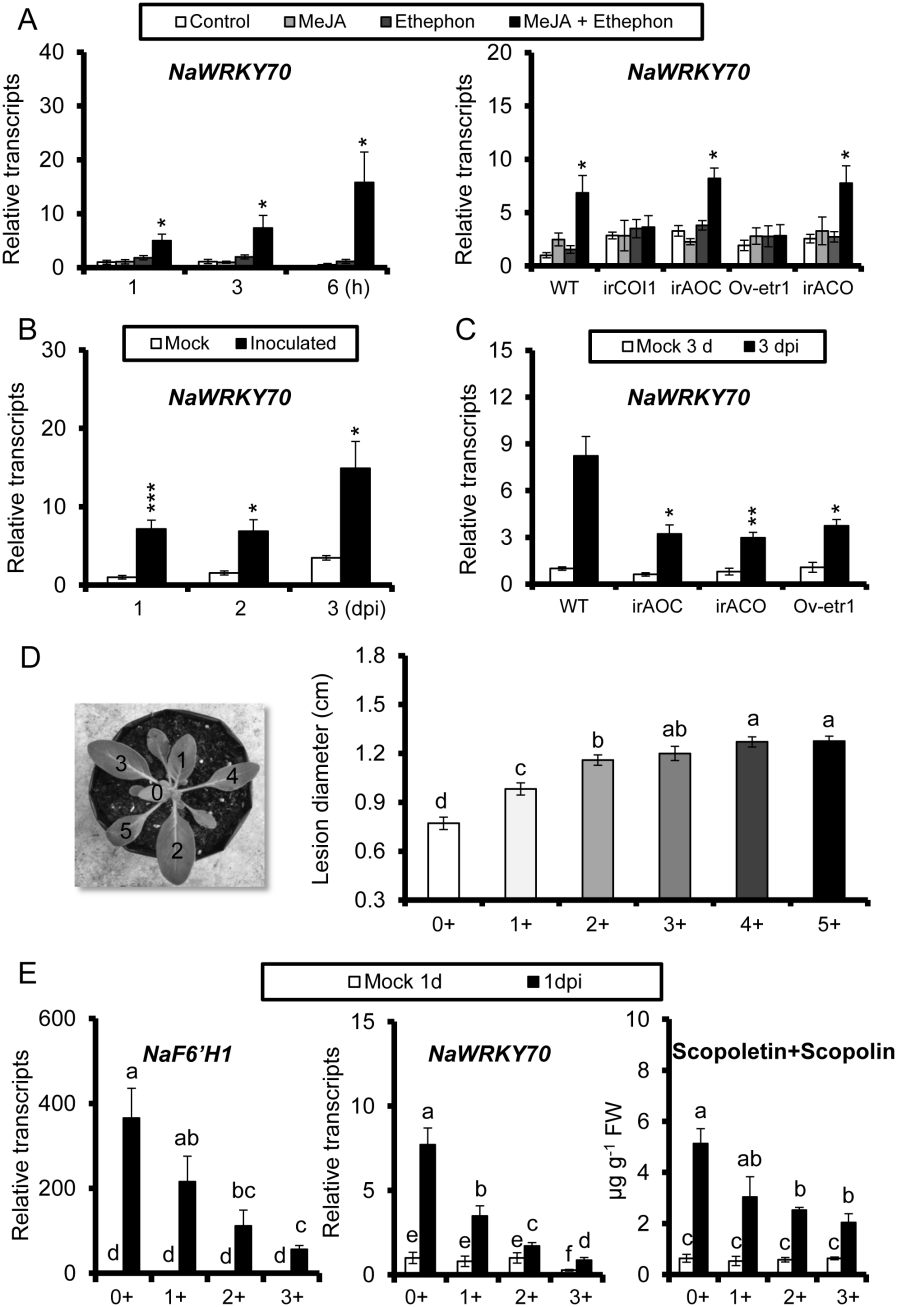
*NaWRKY70* expression induced by *A. alternata* in a JA/ethylene- and age-dependent manner. (A) Left panel: mean (±SE) relative expression levels of *NaWRKY70* were quantified in five biological replicates of 0-leaves by qPCR at 1, 3, and 6 h in response to water, MeJA, ethephon, or co-treatment with MeJA and ethephon (left panel). Asterisks indicate the level of significant differences between control and samples co-treated with MeJA and ethephon at the same time points (Student’s *t*-test: *P<0.05). Right panel: mean (±SE) relative expression levels of *NaWRKY70* in five biological replicates of 0-leaves of WT, irAOC, irACO, and Ov-etr1 plants after treatment with water control, MeJA, ethephon, or co-treatment of MeJA and ethephon at 6 h. Asterisks indicate the level of significant difference between control and MeJA and ethephon co-treated samples with the same lines (Student’s *t*-test: *P<0.05). (B) Mean (±SE) relative *NaWRKY70* expression levels were analysed in five biological replicated of 0-leaves of WT after infection by *A. alternata* at 1, 2, and 3 dpi. Asterisks indicate the level of significant differences between mock and inoculated samples (Student’s *t*-test: *P<0.05, ***P<0.005). (C) Mean (±SE) relative *A. alternata*-elicited expression levels of *NaWRKY70* were measured in five replicates of 0-leaves of WT, irAOC, irACO, and Ov-etr1 plants at 3 dpi. Asterisks indicate the level of significant differences between WT and irAOC, irACO, or Ov-etr1 plants after infection by *A. alternata* at 3 dpi (Student’s *t*-test: *P<0.05, **P<0.01). (D) Mean (±SE) diameter of necrotic lesions (right panel) from 15 biological replicates in differently numbered rosette leaves of WT infected with *A. alternata* for 5 d. Numbering of the leaves (left panel) at different phyllotaxic positions (nodes) on a rosette-stage plant was done according to Sun *et al.* (2014a). The leaf at node 0 (0+) is at the stage of source–sink transition, one phyllotaxic position younger than the first fully expanded leaf (1+). Similarly, the leaf at node 3 (3+) is also one phyllotaxic position younger than the leaf at node 4 (4+). (E) Mean (±SE) relative expression levels of *NaF6ʹH1* and *NaWRKY70* and scopoletin and scopolin levels measured in eight biological replicates of 0-leaves of WT plants at 1 dpi. Different letters indicate significant differences by two-way ANOVA followed by Duncan’s test (*P*<0.05).

We performed qPCR to investigate *NaWRKY70* expression in WT, irAOC, irCOI1, irACO, and Ov-etr1 plants. As expected, *NaWRKY70* transcripts were strongly elicited and gradually increased in leaves co-treated with MeJA and ethephon at 1, 3 and 6 h, but did not differ in leaves treated with MeJA or ethephon alone at all three time points. This synergistic induction of *NaWRKY70* by MeJA and ethephon was reproduced in JA-deficient irAOC and ethylene-reduced irACO leaves, but was abolished in JA-insensitive irCOI1 or ethylene-insensitive Ov-etr1 plants (Fig. 3A).

The transcriptional levels of *NaWRKY70* were also significantly increased in 0-leaves of WT plants after *A. alternata* inoculation at 1, 2, and 3 days post-inoculation (dpi) (Fig. 3B), and this *A. alternata*-induced expression was reduced by 50%, 70%, and 60% in irAOC, irACO, and Ov-etr1 plants, respectively (Fig. 3C), indicating that both JA and ethylene signaling pathways are required for *A. alternata*-induced *NaWRKY70* expression.

Brown spot disease caused by *A. alternata* usually occurs in mature leaves of both *N. tabacum* and *N. attenuata*, but is barely detectable in young leaves (Cheng and Sun, 2001; Sun *et al.*, 2014b). Indeed, we also found that *N. attenuata* leaves became more susceptible to *A. alternata* with increasing leaf maturity in this study (Fig. 3D). This phenomenon was associated with higher *NaF6ʹH1* expression and scopoletin and scopolin production in young leaves (Fig. 3E). Importantly, *NaWRKY70* was also highly expressed in young leaves, but decreased as leaves matured, as did *NaF6ʹH1* expression and scopoletin and scopolin production (Fig. 3E). These results suggest that NaWRKY70 may be responsible for the age-dependent susceptibility to *A. alternata*.

### NaWRKY70 is a key transcription factor for scopoletin and scopolin production and *A. alternata* resistance

**Fig. 4.**
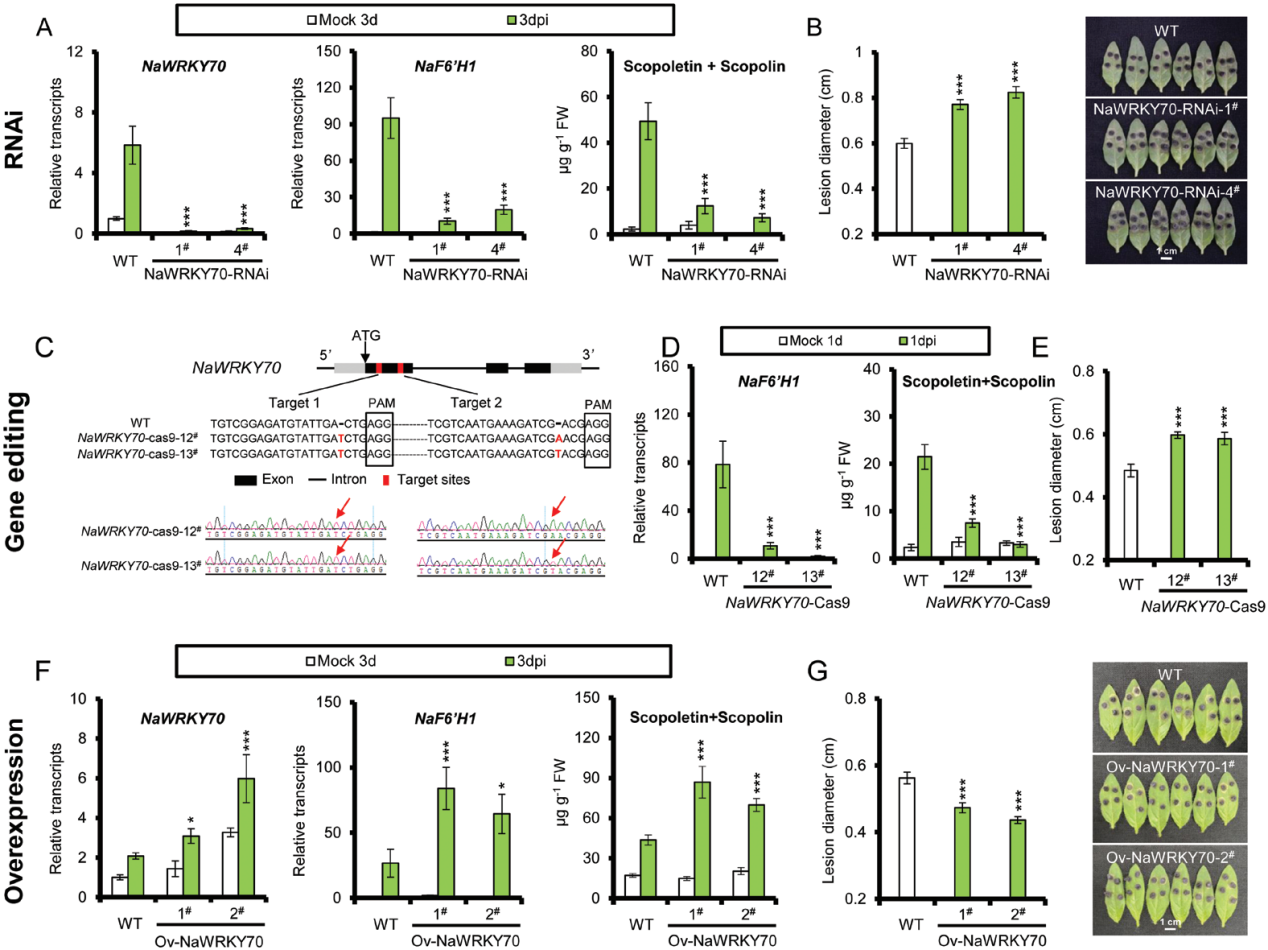
*Alternaria alternata*-induced *NaF6ʹH1* expression, scopoletin and scopolin biosynthesis, and plant resistance to the fungus in *NaWRKY70* silencing lines, knockout mutants and overexpression lines. (A) Mean (±SE) relative *A. alternata*-induced transcriptional levels of *NaWRKY70* (left panel) and *NaF6ʹH1* (middle panel), and scopoletin and scopolin levels (right panel) were measured in five biological replicates of 0-leaves of WT and two independent *NaWRKY70* silencing transgenic plants (NaWRKY70-RNAi-1^#^ and 4^#^) at 3 dpi. Asterisks indicate the level of significant difference between WT and two NaWRKY70-RNAi lines after infection by *A. alternata* (Student’s *t*-test: ***P<0.005). (B) Left panel: Mean (±SE) diameter of necrotic lesions of 15 biological replicates of 0-leaves of WT, NaWRKY70-RNAi-1^#^, and NaWRKY70-RNAi-4^#^ infected with *A. alternata* for 5 d. Right panel: photographs of six representative leaves of each different genotype at 5 dpi. Asterisks indicate the level of significant difference between WT and two NaWRKY70-RNAi lines (Student’s *t*-test: ***P<0.005). (C) Schematic representation of the *NaWRKY70* gene showing the mutation sites generated by CRISPR/Cas9. Two sgRNA sequences of specific targets of *NaWRKY70* are shown in detail, generating *NaWRKY70*-cas9-12^#^ and 13^#^ mutants. Protospacer adjacent motif (PAM) sites are indicated by black squares. The insertion sites of target 1 and target 2 are indicated by red arrows. (D) Mean (±SE) relative *A. alternata*-elicited *NaF6ʹH1* expression levels (left panel) and scopoletin and scopolin levels (right panel) were measured in five biological replicates of 0-leaves of WT and two *NaWRKY70* knockout mutants (*NaWRKY70*-cas9-12^#^ and 13^#^) at 1 dpi. Asterisks indicate the level of significant differences between WT and two *NaWRKY70* mutants after infection by *A. alternata* (Student’s *t*-test: ***P<0.005). (E) Mean (±SE) diameter of necrotic lesions of 15 biological replicates of 0-leaves of WT, *NaWRKY70*-cas-12^#^, and 13^#^ infected with *A. alternata* for 5 d. Asterisks indicate the level of significant difference between WT and two *NaWRKY70* mutants (Student’s *t*-test: ***P<0.005). (F) Mean (±SE) relative transcripts of *NaWRKY70* (left panel) and *NaF6ʹH1* (middle panel), and scopoletin and scopolin levels (right panel) were measured in five biological replicates of 0-leaves from WT and two stable transgenic NaWRKY70 overexpression plants (Ov-NaWRKY70-1^#^ and 2^#^) at 3 dpi. Asterisks indicate the level of significant difference between WT and two NaWRKY70 overexpression plants after infection by *A. alternata* (Student’s *t*-test: *P<0.05, ***P<0.005). (G) Left panel: mean (±SE) diameter of necrotic lesions of 15 biological replicates of 0-leaves of WT, Ov-NaWRKY70-1^#^, and 2^#^ after inoculation with *A. alternata* for 5 d. Right panel: photographs of six representative leaves of each different genotype at 5 dpi. Asterisks indicate the level of significant difference between WT and two NaWRKY70 overexpression plants (Student’s *t*-test: ***P<0.005).

To elucidate the role of NaWRKY70 in *A. alternata* resistance, stable transgenic *NaWRKY70*-silenced lines were generated by RNAi. We selected two independent, homozygous T2 lines of NaWRKY70-RNAi 1^#^ and 4^#^ for further analysis. *Alternaria alternata*-elicited *NaWRKY70* expression in 0-leaves at 3 dpi was successfully silenced in both RNAi lines, with reductions of 95% and 98%, respectively (Fig. 4A). Importantly, *A. alternata*-elicited *NaF6ʹH1* transcripts were also reduced by more than 80%, resulting in 80–90% reductions in scopoletin and scopolin levels at 3 dpi (Fig. 4A). We also found that *NaCCoAOMTs*, key enzyme genes of scopoletin biosynthesis, were down-regulated in two NaWRKY70-RNAi lines (Supplementary Fig. S4). Moreover, NaWRKY70-RNAi plants were more susceptible to *A. alternata* than WT, as much larger lesions developed (Fig. 4B).

To further confirm the results of the RNAi lines, we also generated two *NaWRKY70* knockout mutants (*NaWRKY70*-cas9-12^#^ and 13^#^) using the CRISPR/Cas9 system. These two mutants contained different insertions at the target sites. *NaWRKY70*-cas9-12^#^ had a T insertion in target 1 and an A insertion in target 2, whereas *NaWRKY70*-cas9-13^#^ had a T insertion in target 1 and another T insertion in target 2 (Fig. 4C). Similar to the RNAi lines, both *A. alternata*-induced *NaF6ʹH1* and production of scopoletin and scopolin were severely impaired in the *NaWRKY70* mutants, resulting in plants more susceptible to *A. alternata* (Fig. 4D, E).

We also generated stable transgenic plants overexpressing *NaWRKY70* (Ov-NaWRKY70-1^#^ and 2^#^) by *Agrobacterium*-mediated transformation. *Alternaria alternata*-induced *NaWRKY70* levels were significantly increased, with 149% and 289% of WT levels at 3 dpi, respectively (Fig. 4F). At the same time, *A. alternata*-induced expression levels of *NaF6ʹH1* in the Ov-NaWRKY70 lines were increased to 3.2- and 2.42-fold of that of WT, respectively; and levels of both phytoalexins and *A. alternata* resistance were dramatically increased in Ov-NaWRKY70 lines compared with WT (Fig. 4F, G).

All these results support an important role for NaWRKY70 in the regulation of the phytoalexins scopoletin and scopolin and in *A. alternata* resistance.

### Synergistic induction of scopoletin and scopolin requires intact endogenous jasmonate and ethylene signaling, and is largely dependent on NaWRKY70

**Fig. 5:**
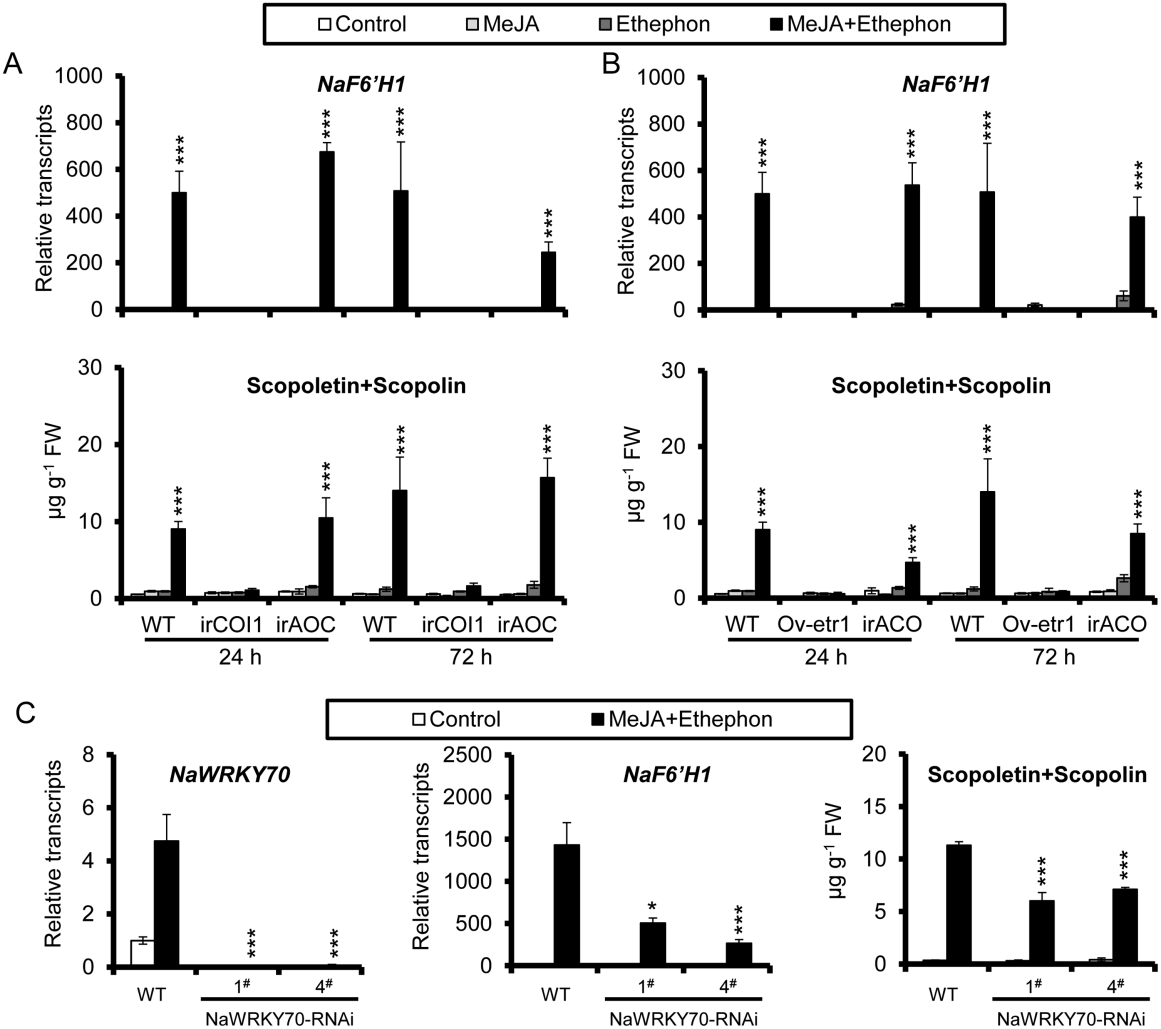
Synergistic induction of scopoletin and scopolin requires intact endogenous JA and ethylene signaling, and is largely dependent on NaWRKY70. (A) Mean (±SE) *NaF6ʹH1* transcript levels (upper panel) and scopoletin and scopolin levels (bottom panel) were measured when treated with water control, MeJA, ethephon, or co-treatment with MeJA and ethephon in five biological replicates of 0-leaves of WT, irCOi1, and irAOC plants at 24 h and 72 h. Asterisks indicate the level of significant difference between control and MeJA and ethephon co-treated samples with the same lines (Student’s *t*-test: ***P<0.005). (B) Mean (±SE) *NaF6ʹH1* transcript levels (upper panel) and scopoletin and scopolin levels (bottom panel) were measured when treated with water control, MeJA, ethephon, or co-treatment with MeJA and ethephon in five biological replicates of rosette leaves of WT, Ov-etr1 plants, and irACO plants at 24 h and 72 h. Asterisks indicate the level of significant difference between control and MeJA and ethephon co-treated samples with the same lines (Student’s *t*-test: ***P<0.005). (C) Mean (±SE) relative transcripts of *NaWRKY70* (left panel) and *NaF6ʹH1* (middle panel), and scopoletin and scopolin levels (right panel) were measured in five biological replicates of 0-leaves from WT and two NaWRKY70-RNAi lines after MeJA and ethephon co-treatment. Asterisks indicate the level of significant difference between WT and two NaWRKY70-RNAi lines after MeJA and ethephon co-treatment (Student’s *t*-test: *P<0.05, ***P<0.005).

To further confirm the role of endogenous JA and ethylene signaling in the synergistic induction of scopoletin and scopolin upon co-treatment with MeJA and ethephon, we analysed the transcriptional levels of *NaF6ʹH1* and scopoletin and scopolin production in WT and transgenic plants of JA-deficient irAOC, JA-insensitive irCOI1, ethylene-reduced irACO, and ethylene-insensitive Ov-etr1. Our results showed that the synergistic induction of both *NaF6ʹH1* transcripts and phytoalexin production by MeJA and ethephon was observed in WT and JA-deficient irAOC leaves, but was abolished in JA-insensitive irCOI1 leaves (Fig. 5A). Similarly, no effect of MeJA and ethephon co-treatment on *NaF6ʹH1* expression and scopoletin and scopolin accumulation was observed in ethylene-insensitive Ov-etr1 plants (Fig. 5B). Thus, our results indicate that intact endogenous JA and ethylene signaling is required not only for *A. alternata*-induced scopoletin and scopolin production, but also for the synergistic induction of scopoletin and scopolin by MeJA and ethephon.

Since *NaWRKY70* expression is synergistically induced by MeJA and ethephon (Fig. 3A), and *NaWRKY70* regulates *A. alternata*-induced scopoletin and scopolin production (Fig. 4), we tested whether *NaWRKY70* is required for the synergistic induction of scopoletin and scopolin by MeJA and ethephon. As expected, the synergistic induction of *NaF6ʹH1*, scopoletin, and scopolin by co-treatment with MeJA and ethephon for 3 d was severely impaired in NaWRKY70-RNAi plants (Fig. 5C). These results suggest that intact endogenous JA and ethylene signaling is required for the synergistic induction of scopoletin and scopolin, and that this synergistic induction is largely dependent on NaWRKY70.

### NaWRKY70 regulates scopoletin and scopolin biosynthesis by directly binding and activating the *NaF6ʹH1* promoter

**Fig. 6.**
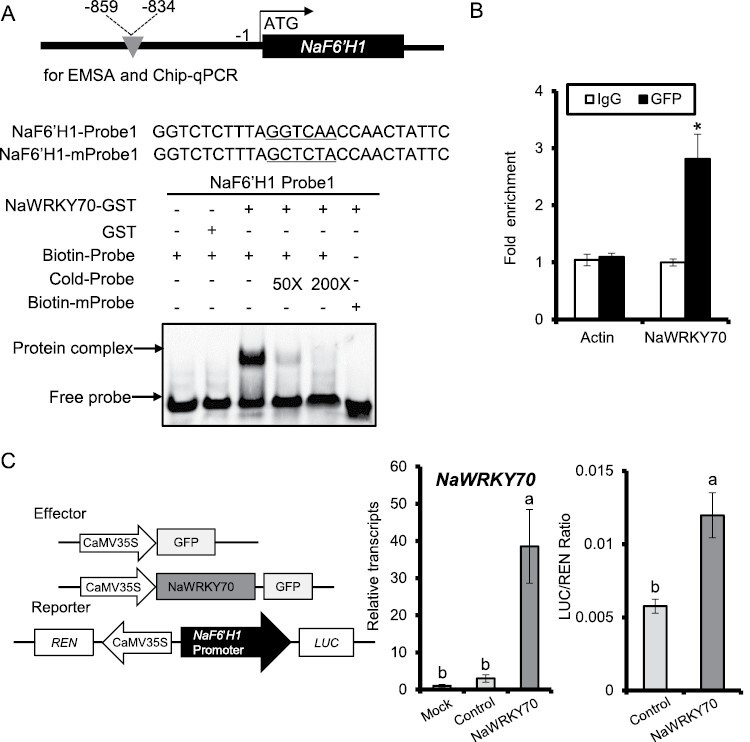
NaWRKY70 binds directly to the promoter of *NaF6ʹH1* and activates its transcriptional activity. (A) Schematic representation of the *NaF6ʹH1* promoter and electrophoretic mobility shift assay (EMSA) result of NaWRKY70 binding to the *NaF6ʹH1* promoter. The sequence and position of probe 1 (and mutant: mProbe 1) in the *NaF6ʹH1* promoter are indicated. EMSA showed that NaWRKY70 protein could specifically bind to probe 1. The mobility shift was abolished by the addition of cold unlabeled probes, and no signals were detected when the mutated probe was used. (B) Detection of *in vivo* binding of NaWRKY70 to the *NaF6ʹH1* promoter by ChIP–qPCR. In NaWRKY70-eGFP transgenic lines, higher levels of *NaF6ʹH1* promoter regions around probe 1 were enriched with GFP antibody. Negative controls were immunoprecipitated with IgG or with GFP antibody but using primers that detect the *Actin* gene. Asterisks indicate the levels of significant differences between samples immunoprecipitated with IgG and anti-GFP (Student’s *t*-test: *P<0.05). (C) Overexpression of *NaWRKY70* led to activation of the *NaF6ʹH1* promoter. Left panel: schematic representation of the effector and reporter constructs used in the transient dual-LUC assays. The effector construct contained the *NaWRKY70* coding sequence driven by the CaMV35S promoter. The reporter construct contained LUC driven by the promoter of *NaF6ʹH1* and REN driven by the CaMV 35S promoter. Middle panel: in the transient dual-LUC assays, mean (±SE) *NaWRKY70* transcript levels were dramatically increased in transient *NaWRKY70* overexpression samples compared with mock (untreated samples) or control (transient overexpression of empty vector samples). Right panel: overexpression of NaWRKY70 could significantly lead to the activation of the *NaF6ʹH1* promoter. Different letters indicate significant differences by two-way ANOVA followed by Duncan’s test (*P*<0.05).

Since NaWRKY70 was required for *A. alternata*-induced *NaF6ʹH1* expression, scopoletin and scopolin accumulation, we hypothesized that the WRKY70 protein might bind directly to the *NaF6ʹH1* promoter. *NaF6ʹH1* has five conserved W-boxes in its promoter region, which could be potential binding sites for WRKYs. We therefore designed biotin-labeled probes containing respectively these five W-boxes for EMSA. NaWRKY70-GST protein was expressed and purified from *E. coli*. EMSA showed that NaWRKY70 protein could only bind to one of these five probes, probe 1 (Supplementary Fig. S5; Fig. 6A). This binding was specific, as binding was attenuated by the addition of 50-fold unlabeled cold probe and abolished by 200-fold cold probe. Importantly, the mutant probe lost its binding activity with NaWRKY70 (Fig. 6A).

We next confirmed the binding of NaWRKY70 to the *NaF6ʹH1* promoter by ChIP–qPCR in transgenic lines overexpressing *NaWRKY70*-*eGFP*. Higher levels of *NaF6ʹH1* promoter regions around probe 1 were enriched with GFP antibody, indicating that NaWRKY70 can bind to the promoter of *NaF6ʹH1 in vivo* via this W-box sequence (Fig. 6B).

NaWRKY70 also transiently activated the *NaF6ʹH1* promoter in a dual-LUC system assay. As shown in Fig. 6C, a significantly higher LUC/REN ratio was detected, indicating that NaWRKY70 activated the *NaF6ʹH1* promoter, thereby increasing LUC activity.

Thus, our data clearly indicate that NaWRKY70 directly binds to the *NaF6ʹH1* promoter and activates *NaF6ʹH1* expression.

### NaEIN3-like and NaEIN3-like1 regulate *NaWRKY70* and *NaF6ʹH1* expression and scopoletin and scopolin production

**Fig. 7.**
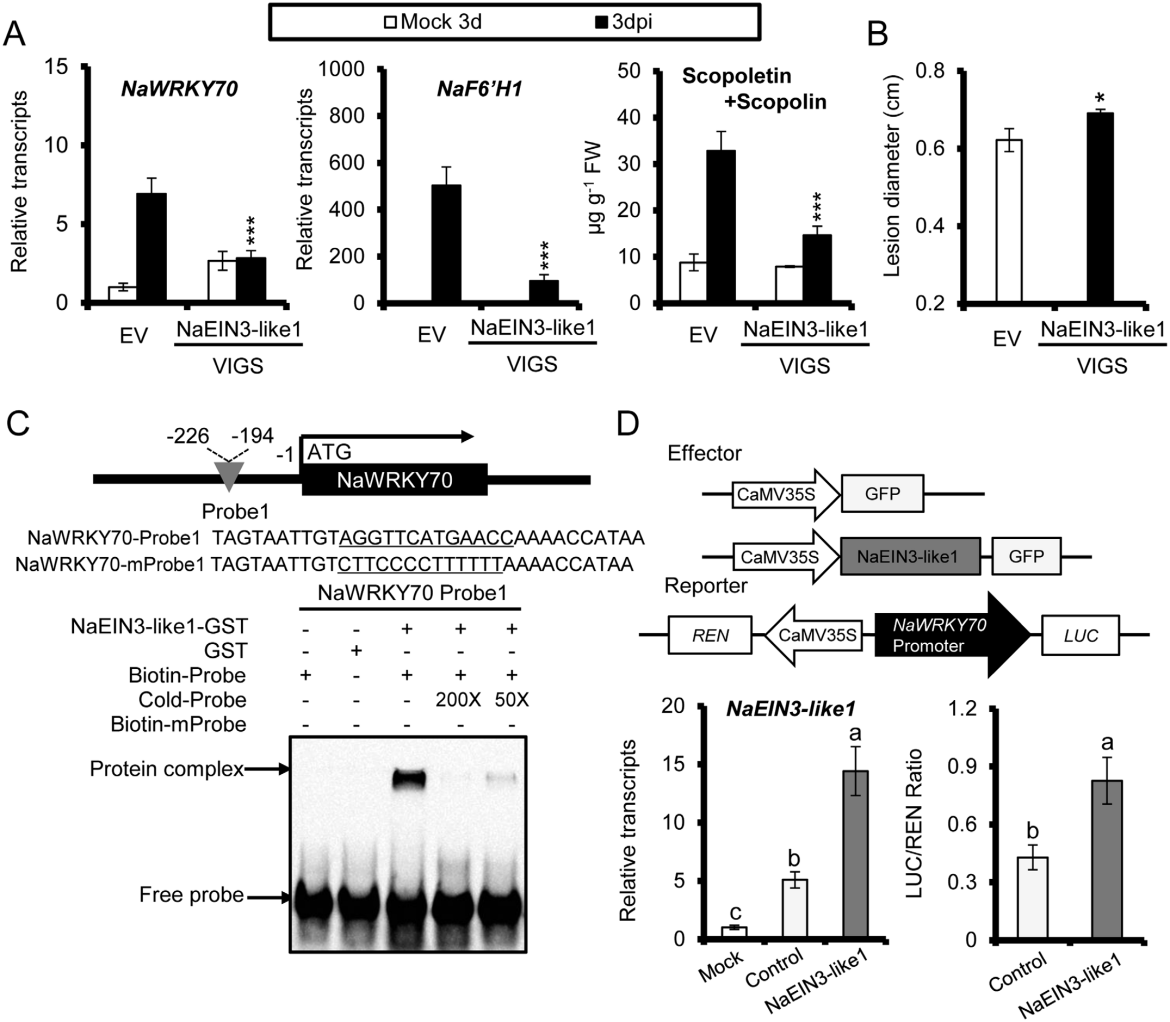
NaEIN3-like1 regulates *NaWRKY70* and *NaF6ʹH1* expression, scopoletin and scopolin production, and plant resistance to *A. alternata.* (A) Mean (±SE) relative expression levels of *NaWRKY70* (left panel) and *NaF6ʹH1* (middle panel), and scopoletin and scopolin levels (right panel) were measured in five biological replicates of young leaves of EV and VIGS NaEIN3-like1 at 3 dpi. Asterisks indicate the level of significant difference between WT and VIGS NaEIN3-like1 after infection by *A. alternata* (Student’s *t*-test: ***P<0.005). (B) Mean (±SE) diameter of necrotic lesions of 15 biological replicates of young leaves of EV and VIGS NaEIN3-like1 infected with *A. alternata* for 5 d. Asterisks indicate the level of significant difference between WT and VIGS NaEIN3-like1 (Student’s *t*-test: *P<0.05). (C) Schematic diagram of the *NaWRKY70* promoter and EMSA result of binding of NaEIN3-like1 to the *NaWRKY70* promoter. Probe 1 was designed from the *NaWRKY70* promoter as indicated. EMSA showed that NaEIN3-like1 protein could specifically bind to probe 1. The mobility shift was suppressed by cold unlabeled probes. (D) Overexpression of *NaEIN3-like1* resulted in activation of the *NaWRKY70* promoter. Upper panel: schematic diagram showing the effector and reporter constructs used in the transient dual-LUC assays. The effector construct contained the *NaEIN3-like1* coding sequence driven by the CaMV35S promoter. The reporter construct contained LUC driven by the promoter of *NaWRKY70* and REN driven by the CaMV 35S promoter. Bottom left panel: in the transient dual-LUC assays, mean (±SE) *NaEIN3-like1* transcript levels were dramatically increased in transient *NaEIN3-like1* overexpression samples compared with mock (untreated samples) or control (transient overexpression of empty vector samples). Bottom right panel: overexpression of *NaEIN3-like1* could significantly lead to activation of the *NaWRKY70* promoter. Different letters indicate significant differences by two-way ANOVA followed by Duncan’s test (*P*<0.05).

The transcription factors EIN3 and EIN3-like1 (EIL1) are two important positive regulators of the ethylene pathway in Arabidopsis. In *N. attenuata*, their homologs, NaEIN3-like and NaEIN3-like1, were highly elicited by *A. alternata* during transcriptome analysis (Supplementary Table 2). When *NaEIN3-like1* was silenced by VIGS, *A. alternata*-elicited *NaEIN3-like1*, *NaF6ʹH1*, and *NaWRKY70* expression, scopoletin and scopolin accumulation, and plant resistance to the fungus, were strongly impaired (Supplementary Fig. S6; Fig. 7A, B). EMSA showed that NaEIN3-like1 could specifically bind to one of the probes designed from the *NaWRKY70* promoter, and this binding was abolished by the addition of 200 times unlabeled cold probe (Fig. 7C). To further analyse the regulatory effect of NaEIN3-like1 protein on *NaWRKY70* expression, we transformed the 35s::NaEIN3-like1 construct in *N. benthamiana*. The LUC/REN ratio was greatly increased compared with the negative controls (Fig. 7D), indicating that NaEIN3-like1 could activate *LUC* expression under the control of the *NaWRKY70* promoter.

When *NaEIN3-like* was silenced by VIGS, *A. alternata*-elicited *NaEIN3-like*, *NaF6ʹH1*, and *NaWRKY70* expression, scopoletin and scopolin accumulation, and plant resistance to the fungus, were also greatly reduced (Supplementary Fig. S7A, B). However, NaEIN3-like could not bind to four predicted probes designed from the *NaWRKY70* promoter (Supplementary Fig. S7C), suggesting that NaEIN3-like regulates *NaF6ʹH1* expression indirectly.

Taken together, our results indicate that (i) both NaEIN3-like and NaEIN3-like1 positively regulate *NaWRKY70* expression, and (ii) at least NaEIN3-like1 directly binds and activates *NaWRKY70* expression.

### NaMYC2s are involved in the expression of NaWRKY70 and *NaF6ʹH1*, the production of scopoletin and scopolin, and plant resistance to *A. alternata*

**Fig. 8.**
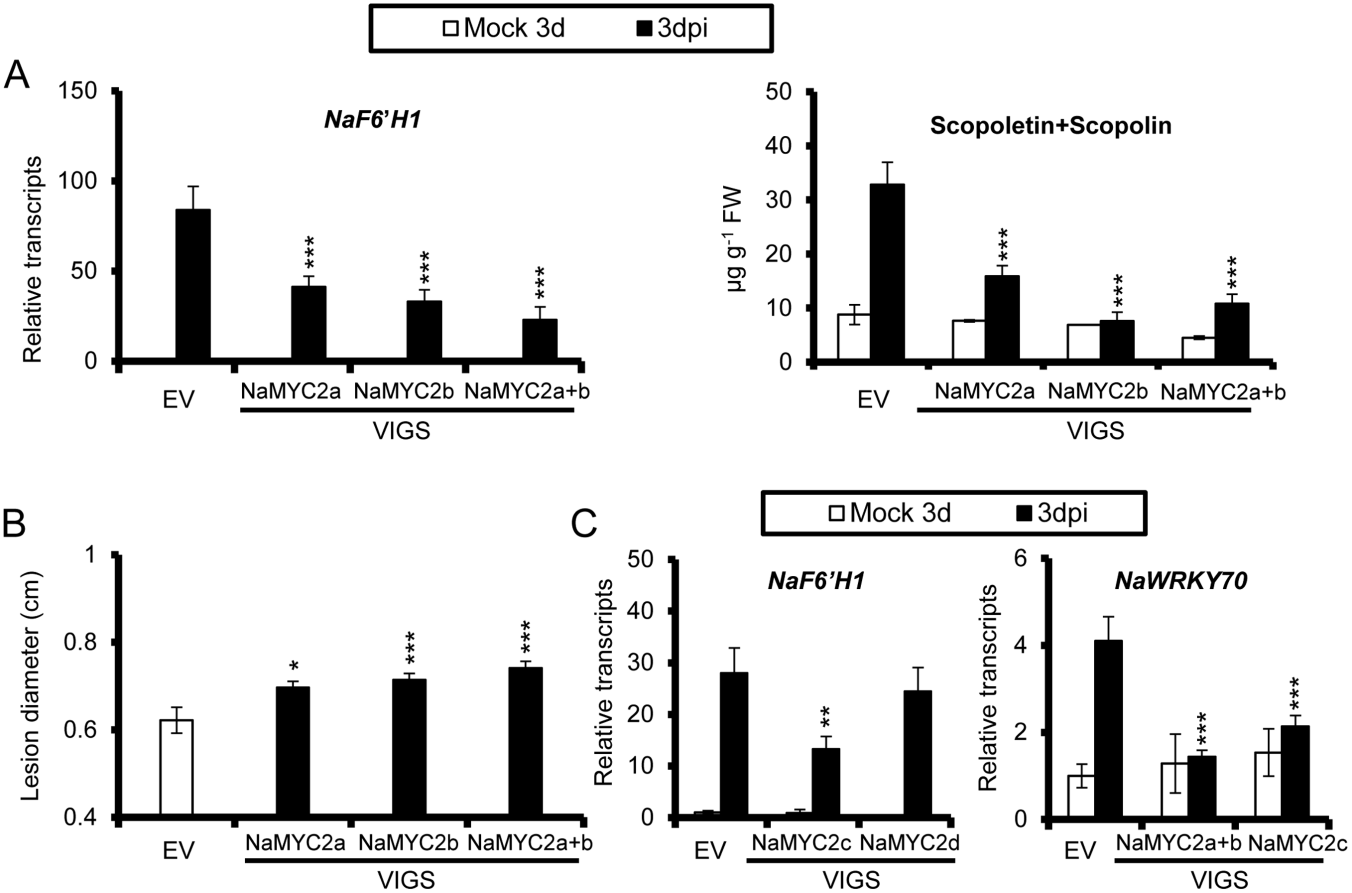
NaMYC2s are involved in the expression of *NaWRKY70* and *NaF6ʹH1*, the production of scopoletin and scopolin, and *A. alternata* resistance. (A) Mean (±SE) relative *NaF6ʹH1* transcripts (left panel), and scopoletin and scopolin levels (right panel) were measured in five biological replicates of young leaves of EV, VIGS NaMYC2a, VIGS MYC2b, and VIGS MYC2a+b plants at 3 dpi. Asterisks indicate the level of significant difference between EV and VIGS plants after infection by *A. alternata* (Student’s *t*-test: ***P<0.005). (B) Mean (±SE) diameter of necrotic lesions of 15 biological replicates of young leaves of EV, VIGS NaMYC2a, VIGS MYC2b, and VIGS MYC2a+b infected with *A. alternata* for 5 d. Asterisks indicate the level of significant difference between EV and VIGS plants (Student’s *t*-test: *P<0.05, ***P<0.005). (C) Mean (±SE) relative *NaF6ʹH1* transcripts were measured in five biological replicates of young leaves of EV, VIGS NaMYC2c, and VIGS MYC2d at 3 dpi (left panel). Mean (±SE) relative *NaWRKY70* transcripts were measured in five biological replicates of young leaves of EV, VIGS MYC2a+b, and VIGS MYC2c at 3 dpi (right panel). Asterisks indicate the level of significant difference between EV and VIGS plants after infection by *A. alternata* (Student’s *t*-test: **P<0.01, ***P<0.005).

Previous studies indicated that the transcription factor MYC2 is a master regulator in JA signaling that modulates downstream target genes (Zhai and Li, 2019). There are four MYC2 homologs, NaMYC2a, NaMYC2b, NaMYC2c, and NaMYC2d, in the *N. attenuata* genome (Supplementary Table 2; Supplementary Fig. S8). Protein sequence analysis showed that NaMYC2a and NaMYC2b clustered together with the highest similarity (Supplementary Fig. S8). We silenced all the MYC2 homologs separately by VIGS (Supplementary Fig. S9). In VIGS NaMYC2a, VIGS NaMYC2b, and VIGS NaMYC2a+b plants, *A. alternata*-induced *NaF6ʹH1* expression, scopoletin and scopolin production, and plant resistance were strongly reduced (Fig. 8A, B). In addition, *A. alternata*-induced transcripts of *NaF6ʹH1* were also impaired in NaMYC2c-silenced plants, whereas they were not altered in VIGS NaMYC2d plants (Fig. 8C). Interestingly, *A. alternata*-elicited *NaWRKY70* transcripts were decreased in VIGS NaMYC2a+b and VIGS NaMYC2c plants (Fig. 8C). Sequence analysis revealed that the *NaWRKY70* promoter contains several G-boxes (CACGTG) or E-boxes (CANNTG) that are targeted by MYC2. However, EMSA assays did not support the idea that NaMYC2a–GST, NaMYC2b–GST, and NaMYC2c–GST proteins could bind to these boxes (Supplementary Fig. S10).

These results indicate that NaMYC2a, NaMYC2b, and NaMYC2c are required for *A. alternata*-induced *NaWRKY70* and *NaF6ʹH1* expression.

## Discussion

### Synergistic induction of scopoletin and scopolin by jasmonate and ethylene signaling

Phytoalexins are an important part of the defense arsenal of plants after pathogen attack, and are thus under the control of a multi-layered network of signaling pathways. Camalexin, a major phytoalexin in Arabidopsis, is cooperatively regulated by CPK5/CPK6 and MPK3/MPK6-mediated phosphorylation after *Botrytis cinerea* infection (Zhou *et al.*, 2020). In maize, the accumulation of kauralexin and zealexin is synergistically induced by co-treatment with JA and ethylene (Fu *et al.*, 2018).

We have previously shown that *A. alternata*-elicited scopoletin and scopolin production is dependent on JA and ethylene signaling pathways in *N. attenuata* (Sun *et al.*, 2014b; Sun *et al.*, 2017), while the regulatory mechanisms of these two signaling pathways remained unknown. In this study, we demonstrated the synergistic regulation of scopoletin and scopolin biosynthesis by JA and ethylene signaling. We observed that scopoletin and scopolin were dramatically induced in *N. attenuata* leaves after co-treatment with MeJA and ethephon, but not by MeJA or ethephon alone (Fig. 1). This synergistic induction was conserved in the genus *Nicotiana*, and can also be observed in the lamina of young and mature leaves and the mid-rib of young and mature leaves of *N. attenuata* (Fig. 1; Supplementary Fig. S2).

When co-treated with MeJA and ethephon, the exogenously applied MeJA is demethylated to JA in the leaves with the help of methyl jasmonate esterase (Wu *et al.*, 2008), and ethephon releases ethylene. Thus, we also observed the synergistic induction of scopoletin and scopolin in JA-deficient irAOC and ethylene-reduced irACO plants. However, the synergistic induction was abolished in irCOI1 (JA-insensitive) or Ov-etr1 (ethylene-insensitive) plants (Fig. 5), suggesting that intact endogenous JA and ethylene signaling pathways are required.

When *N. attenuata* leaves were challenged by *A. alternata*, they produced high levels of JA and ethylene, leading to the synergistic induction of scopoletin and scopolin. This may explain why *A. alternata* could not induce *NaF6ʹH1* expression at all in JA-deficient irAOC plants (Sun *et al.*, 2014b). In ethylene-reduced irACO and ethylene-insensitive Ov-etr1 plants, *A. alternata*-elicited *NaF6ʹH1* expression is strongly impaired (Sun *et al.*, 2017), indicating that ethylene signaling is required for *A. alternata*-induced scopoletin and scopolin production. Our results show that similar to *NaMLP* (Yang *et al.*, 2023), *A. alternata*-induced scopoletin and scopolin biosynthesis is regulated by JA and ethylene signaling in a synergistic manner, thus providing us with a perfect example of phytoalexins synergistically regulated by two different phytohormone signaling pathways. However, it remains to be investigated whether or not scopoletin and scopolin are fully dependent on the synergistic induction of JA and ethylene signaling.

### NaWRKY70 is required for synergistic induction of phytoalexins by jasmonate and ethylene signaling and *A. alternata* resistance

Many WRKY transcription factors have been reported to be involved in plant defense responses. In Arabidopsis, AtWRKY33 acts as a master transcription factor to directly regulate the expression of the camalexin biosynthesis genes *PAD3* and *CYP71A13* for defense against *B. cinerea* (Mao *et al.*, 2011), and AtWRKY70 has been identified as a key node regulator in the antagonistic regulation of salicylic acid- and JA-mediated defense response (Li *et al.*, 2004). In this study, through combination of RNA-sequencing, co-expression, and functional analysis we demonstrated that NaWRKY70 plays an essential role in the synergistic induction of scopoletin and scopolin by JA and ethylene signaling and by *A. alternata* infection (Figs 4, 5C). Further results showed that NaWRKY70 is essential for *A. alternata*-induced scopoletin and scopolin production by directly binding and activating the *NaF6ʹH1* promoter (Fig. 6; Supplementary Fig. S4).

### NaWRKY70 is associated with age-dependent susceptibility to *A. alternata*

In many plant–pathogen interaction systems, the resistance to pathogens usually depends on the developmental age at which the host plants are infected. Some plants are more susceptible to disease at an early developmental stage and become more resistant as they mature; for example, rice and tobacco plants are more susceptible to *Xanthomonas oryzae* and *Phytophthora nicotianae*, respectively, at the seedling stage. However, *Nicotiana* plants are more resistant to *A. alternata* at the seedling stage, whereas they became susceptible at maturity (Cheng and Sun, 2001; Sun *et al.*, 2014a, b). The mechanism behind this age-dependent susceptibility to *A. alternata* is currently unclear. Previously, we showed that young source–sink transition leaves usually accumulated higher levels of JA, capsidiol, and scopoletin and scopolin than those of mature +3 leaves (Sun *et al.*, 2014a, b; Song *et al.*, 2019). Here, we also found that the pattern of *A. alternata*-induced *NaWRKY70* expression was very similar to that of *NaF6ʹH1* expression and scopoletin and scopolin accumulation. They were all highly elicited in young leaves, but this induction decreased as the leaves matured (Fig. 3D, E). Considering the fact that NaWRKY70 functions as a key mediator of the synergistic induction of scopoletin and scopolin by JA and ethylene signaling, we propose that NaWRKY70 is a potential regulatory node of age and plant defense response to *A. alternata* in *Nicotiana* species.

### NaWRKY70 integrates signaling from jasmonate and ethylene pathways in scopoletin and scopolin biosynthesis

Although JA and ethylene signaling are usually associated with plant defense against necrotrophic pathogens and insect herbivores, complicated modes of interaction between JA and ethylene have been reported. For example, ethylene strongly suppresses JA-induced nicotine biosynthesis in *Nicotiana* species (Shoji *et al.*, 2000). However, JA and ethylene also act synergistically to induce the biosynthesis of the phytoalexins kauralexin and zealexin in maize (Fu *et al.*, 2018) and scopoletin and scopolin in *Nicotiana* species in this study.

In Arabidopsis, *PDF1.2* is the best known defense gene that is synergistically regulated by JA and ethylene signaling through ERF1 (Lorenzo *et al.*, 2003). Later, JAZ proteins were shown to interact with EIN3/EIL1 to suppress their regulation of *ERF1* expression. JA enhances the transcriptional regulation of *ERF1* by EIN3/EIL1 by removing of JAZs, whereas ethylene stimulates *EIN3*/*EIL1* expression and increases their protein levels (Zhu *et al.*, 2011). Thus, EIN3/EIL1 has been implicated as the molecular link in the synergistic induction of *PDF1.2* by JA and ethylene signaling.

In maize, *ZmWRKY79* has been proposed to be involved in the synergistic induction of kauralexin and zealexin by JA and ethylene signaling. Similarly, we found that the synergistic induction of scopoletin and scopolin by JA and ethylene signaling is largely dependent on NaWRKY70, suggesting that NaWRKY70 may also act as a convergence node of JA and ethylene signaling in the regulation of phytoalexin biosynthesis. Of course, it will be very interesting to test whether the mechanism found in Arabidopsis also operates in *N. attenuata*.

Currently, it is unknown how JA and ethylene signaling regulate NaWRKY70. However, we have found that NaWRKY70 expression is regulated by key factors in JA and ethylene signaling. *NaEIN3-like* and *NaEIN3-like1*, the homologs of EIN3 and EIL1 genes in Arabidopsis, were both induced in response to *A. alternata* (Fig. 7A; Supplementary Fig. S7A)*. Alternaria alternata*-induced *NaF6ʹH1* and *NaWRKY70* transcripts, scopoletin and scopolin levels, and resistance to the fungus were all impaired in *NaEIN3-like-* or *NaEIN3-like1*-silenced plants (Fig. 7A, B; Supplementary Fig. S7A, B). Interestingly, we found that NaEIN3-like1 can directly bind to the *NaWRKY70* promoter and activate its transcriptional activity (Fig. 7C). Thus, we have demonstrated a novel role for NaEIN3-like1 in response to *A. alternata*, acting as an upstream regulator to positively influence *NaWRKY70* expression, thereby improving *NaF6ʹH1* expression and scopoletin and scopolin accumulation.

In our previous study, we showed that NaMYC2a is involved in scopoletin and scopolin biosynthesis (Sun *et al.*, 2014b). Here, we found that *A. alternata*-induced transcripts of *NaWRKY70* and *NaF6ʹH1* were significantly blocked in VIGS NaMYC2a, VIGS NaMYC2b, and VIGS NaMYC2c plants but not in VIGS NaMYC2d plants (Fig. 8). These results indicated that NaMYC2s can enhance *NaF6ʹH1* expression and scopoletin and scopolin biosynthesis through the positive regulation of *NaWRKY70* expression. We further showed that NaMYC2a, NaMYC2b, and NaMYC2c proteins are unlikely to bind to G-boxes or E-boxes in the *NaWRKY70* promoter (Supplementary Fig. S10). Therefore, it is likely that NaMYC2s activate another unidentified regulator to indirectly regulate *NaWRKY70* expression.

Although we have shown that *NaWRKY70* expression is regulated by *NaEIN3-like*s and *NaMYC2*s, it is currently unclear how *NaWRKY70* expression is synergistically regulated by these two signaling pathways and why phytoalexins cannot be induced by single treatment with MeJA or ethephon. Clearly, more work is needed to understand the mechanism by which NaWRKY70 works.

**Fig. 9.**
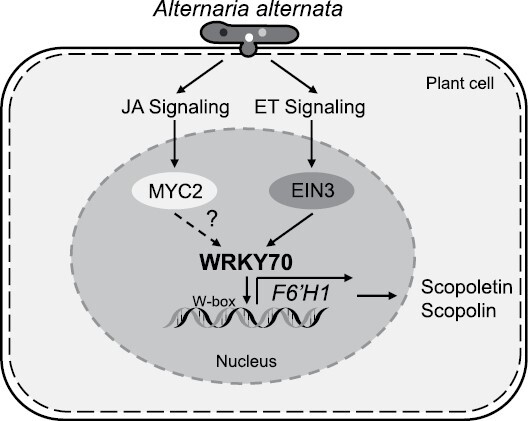
A working model of NaWRKY70-mediated phytoalexin biosynthesis synergistically regulated by JA and ethylene signaling during *A. alternata* infection in *N. attenuata.* In *N. attenuata* plants, both JA and ethylene signaling pathways are activated by *A. alternata*, and synergistically control the biosynthesis of the phytoalexins scopoletin and its β-glycoside form, scopolin, and the expression of their key enzyme gene *NaF6ʹH*. This synergistic induction of phytoalexins by JA and ethylene signaling is largely dependent on NaWRKY70. NaWRKY70 directly binds and activates *NaF6ʹH1* to control scopoletin and scopolin production. Meanwhile, JA and ethylene signaling regulate the expression of *NaWRKY70* through NaMYC2s, NaEIN3-like, and NaEIN3-like1.

Taken altogether, our data uncovered that the phytoalexins scopoletin and scopolin are synergistically regulated by JA and ethylene signaling pathways, and we demonstrate that NaWRKY70 is a key regulator mediating this regulation (Fig. 9). It likely integrates signals from JA and ethylene signaling to control *NaF6ʹH1* expression and phytoalexin biosynthesis. During *A. alternata* infection, both ethylene and JA signaling are activated. NaEIN3-like1 binds directly to the *NaWRKY70* promoter and activates *NaWRKY70* expression. Meanwhile, NaEIN3-like and NaMYC2s also regulate *NaWRKY70* expression, but indirectly. Finally, NaWRKY70 binds directly to the *NaF6ʹH1* promoter and activates *NaF6ʹH1* expression, thereby increasing scopoletin and scopolin biosynthesis. Thus, our findings provide a perfect example of a defense response regulated by JA and ethylene signaling in a synergistic manner, and extend our understanding of phytohormone-regulated defense responses in *Nicotiana* species to *A. alternata*.

## Supplementary data

The following supplementary data are available at *JXB* online.

Fig. S1. Measurement of the *C*_t_ values of *actin*, *Elongation factor 1-alpha*, and *60S ribosomal protein* in leaf samples with different treatments.

Fig. S2. Synergistic induction of scopoletin and scopolin in mid-rib of young leaves, lamina and mid-rib of mature leaves by MeJA and ethephon.

Fig. S3. Protein sequence alignment of NaWRKY70 and its homologs and nuclear localization of NaWRKY70.

Fig. S4. Silencing of *NaWRKY70* impairs *A. alternata*-induced expression of *NaCCoAOMTs*.

Fig. S5. EMSA results showing the binding of NaWRKY70 to one of the five probes designed from the *NaF6ʹH1* promoter.

Fig. S6. The silencing efficiency of *NaEIN3-like1* in VIGS NaEIN3-like1 plants.

Fig. S7. *NaEIN3-like* is required for *A. alternata*-induced transcripts of *NaWRKY70* and *NaF6ʹH1*, scopoletin and scopolin production, and plant resistance to *A. alternata*.

Fig. S8. Protein sequence alignment of NaMYC2a, NaMYC2b, NaMYC2c, and NaMYC2d.

Fig. S9. *NaMYC2a*, *NaMYC2b*, *NaMYC2c*, and *NaMYC2d* were all successfully silenced by VIGS.

Fig. S10. NaMYC*2*a, NaMYC*2*b, and NaMYC*2*c cannot bind to the three probes designed from the *NaWRKY70* promoter.

Table S1. All primers used in this study.

Table S2. *Alternaria alternata*-induced *NaMYC*s and *NaEIN3*, and the top 16 transcription factors synergistically induced by MeJA and ethephon.

erad415_suppl_Supplementary_Tables_S1-S2

erad415_suppl_Supplementary_Figure_S1-S10

## Data Availability

The authors confirm that the data supporting the findings of this study are available within the article and its supplementary data.
