# Supplementary material for: Synergistic induction of phytoalexins in Nicotiana attenuata by jasmonate and ethylene signaling mediated by NaWRKY70
Source: J Exp Bot. 2023 Oct 23;75(3):1063–80. doi: 10.1093/jxb/erad415 (PMC10837013; doi:10.1093/jxb/erad415)
Supplement: erad415_suppl_Supplementary_Figure_S1-S10 [file erad415_suppl_supplementary_figure_s1-s10.pdf]

Synergistic induction of phytoalexins in *Nicotiana attenuata* by JA and ethylene signaling mediated by NaWRKY70

Na Song[1, 2, 3] and Jinsong Wu[1*]

[1]Yunnan Key Laboratory for Wild Plant Resources, Kunming Institute of Botany, Chinese Academy of Sciences, Kunming, 650201, China

[2]University of Chinese Academy of Science, Beijing 10049, China

[3] Yunnan Key Laboratory for Fungal Diversity and Green Development, Kunming Institute of Botany, Chinese Academy of Sciences, Kunming, 650201, China

Running title: Synergistic induction of scopoletin mediated by NaWRKY70

Supplementary Figures

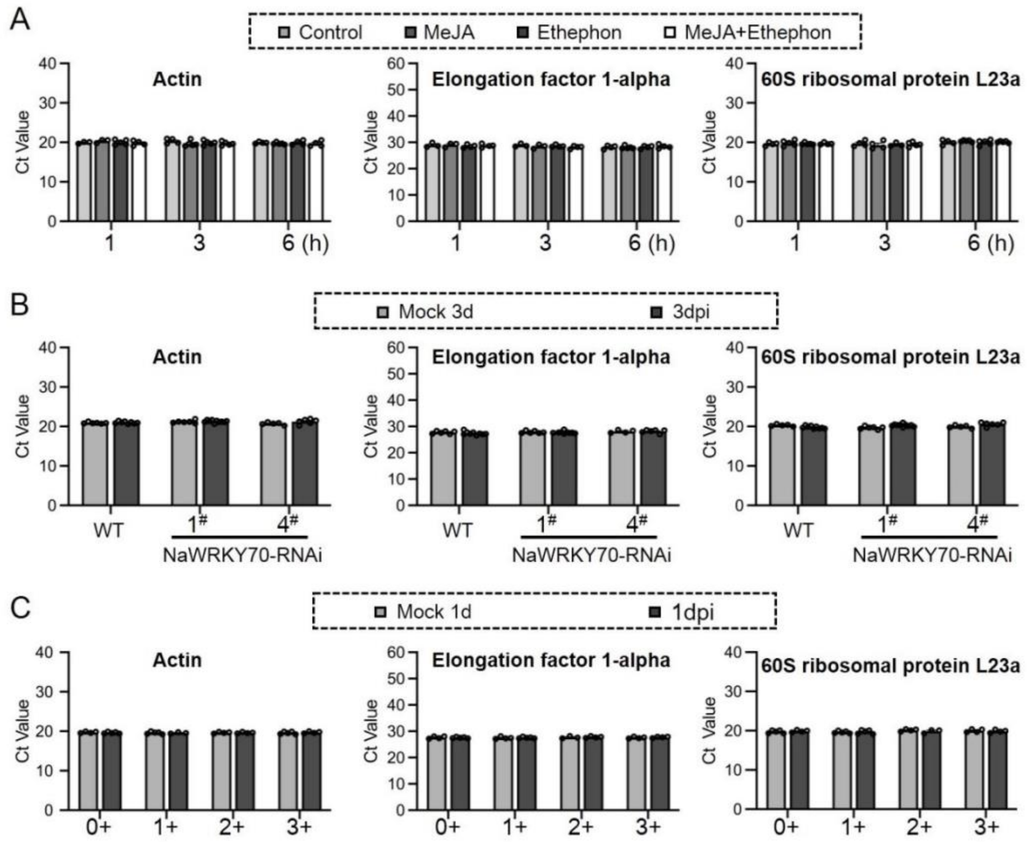

Supplementary Fig. S1 .Measurement of the Ct values of *Actin*, *Elongation factor 1-alpha* and *60S ribosomal protein* in leaf samples with different treatments.

Ct values of *Actin*, *Elongation factor 1-alpha* and *60S ribosomal protein* were determined by qPCR in 0 leaves at 6 h after treatments of water control, MeJA, ethephon, and co-treatments of MeJA and ethephon **(A)**, 0 leaves of WT and two independent *NaWRKY70* silencing transgenic plants (NaWRKY70-RNAi-$1^{\#}$ and $4^{\#}$) at 3 dpi **(B)** and differently numbered rosette leaves of WT at 1 dpi **(C)**.

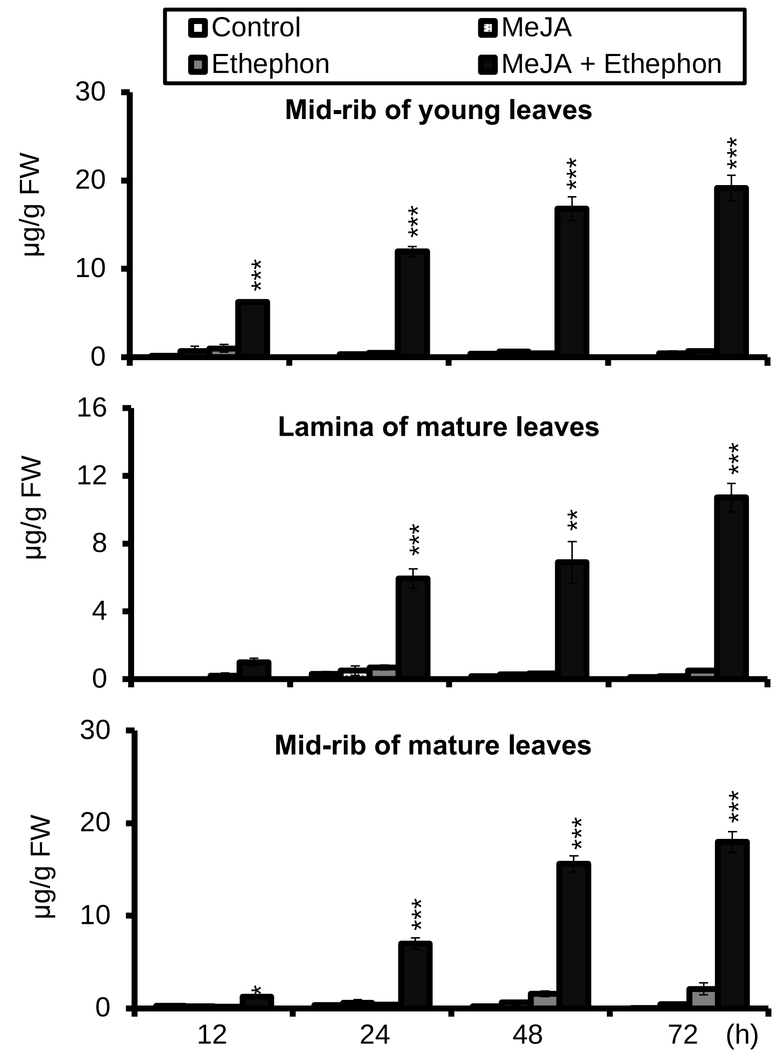

Supplementary Fig. 2. Synergistic induction of scopoletin and scopolin in mid-rib of young leaves, lamina and mid-rib of mature leaves by MeJA and ethephon.

Mean (±SE) scopoletin and scopolin levels were determined in five biological replicates of the mid-rib young leaves, lamina and mid-rib of mature leaves at 12, 24, 48 and 72 h after applying with control water, MeJA, ethephon, and co-treated with MeJA and ethephon. Asterisks indicate the level of significant differences between control and MeJA and ethephon co-treated samples with the same time points (Student's *t*-test: **, $P<0.01$; ***, $P<0.005$).

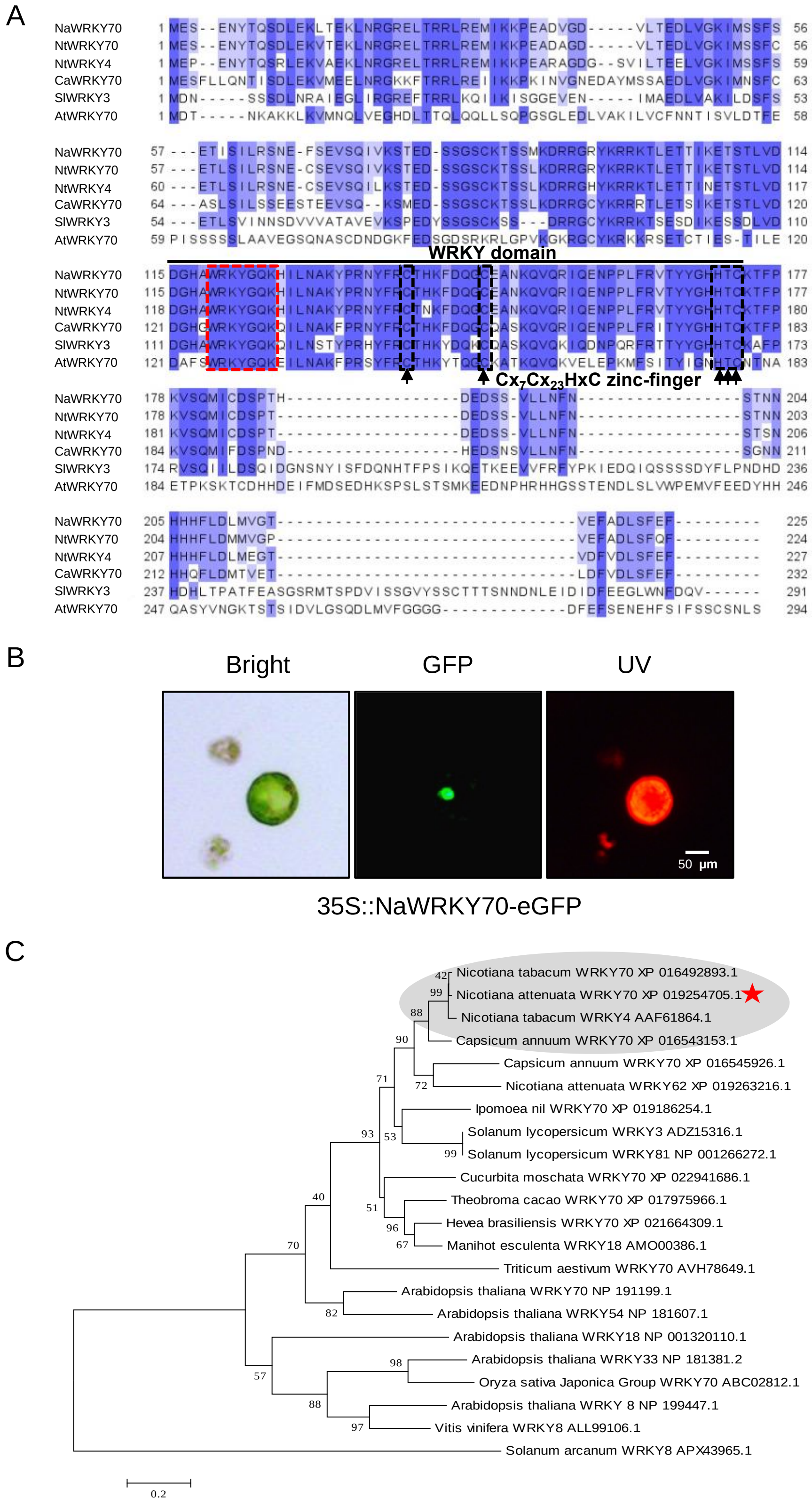

Supplementary Fig. S3. Protein sequence alignment of NaWRKY70 and its homologues and nuclear localization of NaWRKY70.

(A) Sequence alignment of NaWRKY70 and its homologs (including *Nicotiana tabacum* WRKY70, *Nicotiana tabacum* WRKY4, *Capsicum annuum* WRKY70, *Solanum*

lycopersicum WRKY3 and *Arabidopsis thaliana* WRKY70). NaWRKY70 encoded a peptide of 225 amino acids, containing a conserved WRKYGQK domain (red square) and a Cx7Cx23HxC zinc-finger (black square).

(B) Subcellular localization of WRKY70. When the 35S::NaWRKY70-eGPF fusion protein was transformed into the *N. attenuata* leaf protoplasts, strong GFP fluorescence was observed in the nucleus.

(C) Phylogenetic tree of NaWRKY70 with other WRKY transcription factors from different species using the MEGA software by the neighbor-joining (NJ) program. NaWRKY70 was shown by red star. The grey part indicated NaWRKY70, NtWRKY70 and NtWRKY4 were clustered together.

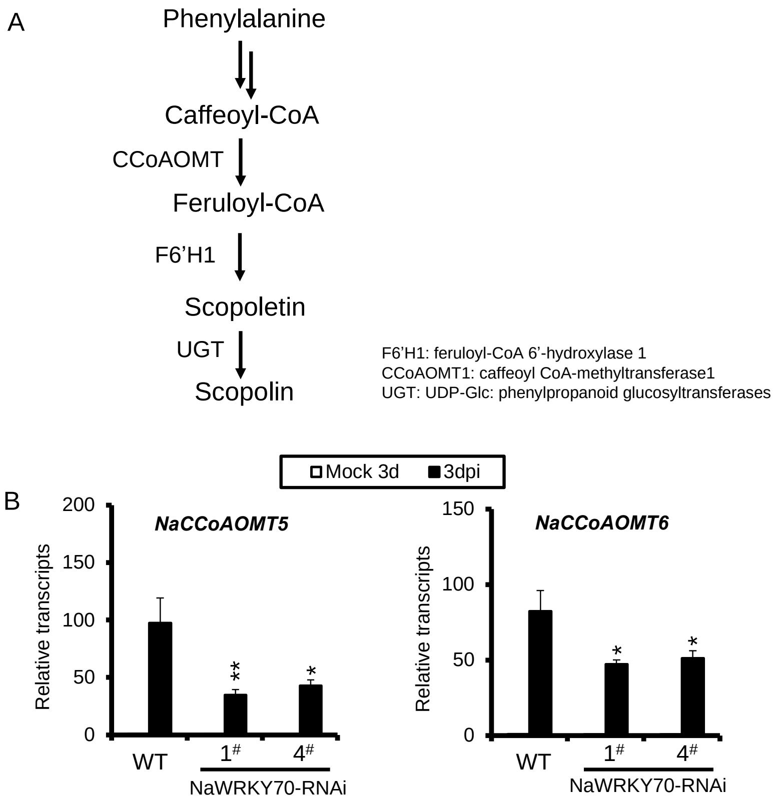

Supplementary Fig. S4. Silencing of *NaWRKY70* impairs *A. alternata*-induced expression of *NaCCoAOMTs*.

(A) Schematic diagrams of scopoletin and scopolin biosynthetic pathway. Scopolin, a β-glycoside form of scopoletin by *UDP-Glc:glucosyltransferases* (*UGTs*). The *CCoAOMT* and *F6'H1* were key genes of scopoletin and scopolin biosynthesis.

(B) Mean (±SE) relative expression levels of *NaCCoAOMT5* and *NaCCoAOMT6* were measured by qPCR in five biological replicates of WT and two independent *NaWRKY70* silencing transgenic plants (NaWRKY70-RNAi-1# and 4#) at 3 dpi. Asterisks indicate the level of significant differences between WT and two NaWRKY70-RNAi plants after infection by *A. alternata* (Student's *t*-test: *, P<0.05; **, P<0.01).

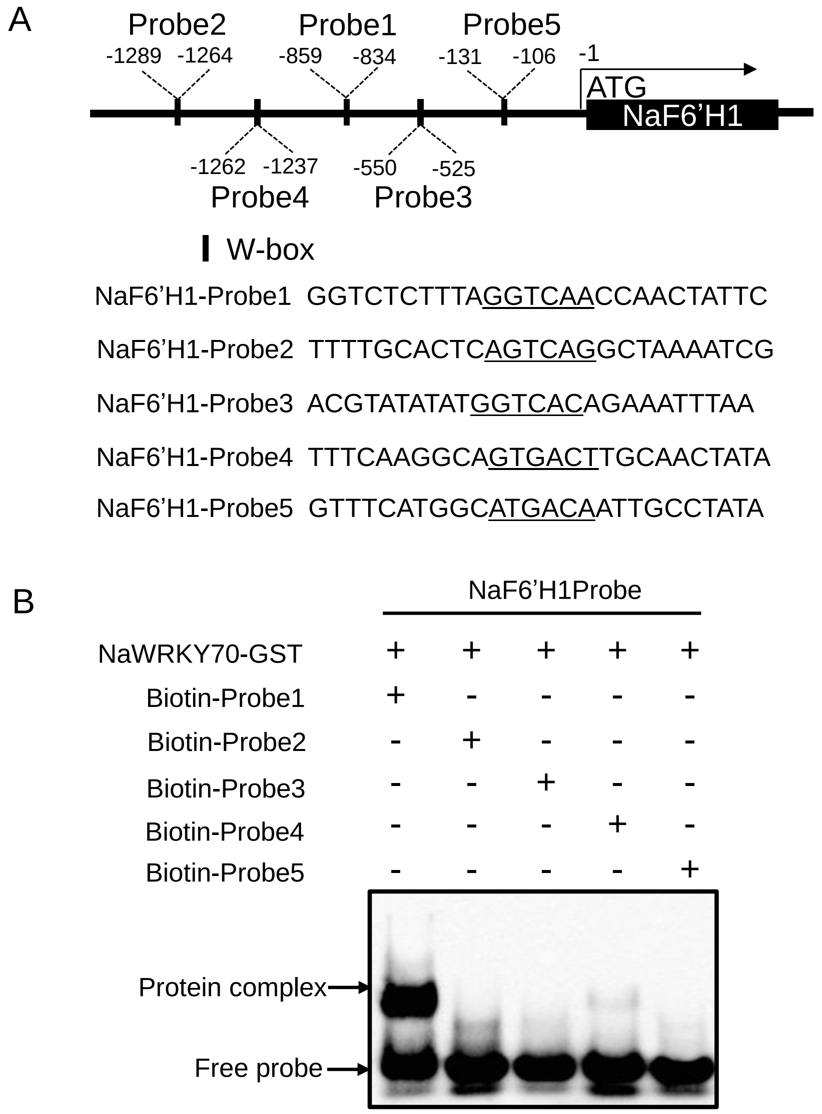

Supplementary Fig. S5. EMSA results showing the binding of NaWRKY70 to one of the five probes designed from the *NaF6'H1* promoter.

(A) Schematic diagrams of the *NaF6'H1* promoter and sequences of five candidate probes. The black lines indicated the positions of predicted W-boxes in the *NaF6'H1* promoter.

(B) EMSA results demonstrated that NaWRKY70 protein could directly bind to Probe1 but not to other probes.

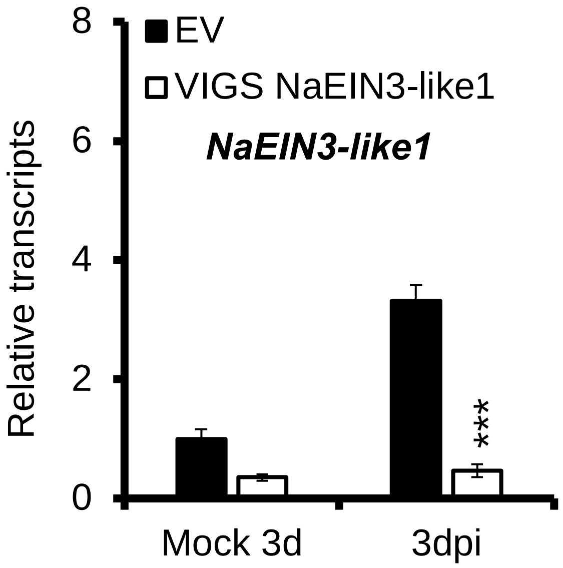

Supplementary Fig. S6. The silencing efficiency of *NaEIN3-like1* in VIGS NaEIN3-like1 plants.

Mean (±SE) relative expression levels of *NaEIN3-like1* were measured in five biological replicated young leaves of EV and VIGS NaEIN3-like1 plants at 3 dpi. Asterisks indicate the level of significant differences between WT and VIGS NaEIN3-like1 plants after infection by *A. alternata* (Student's *t*-test: ***, P<0.005).

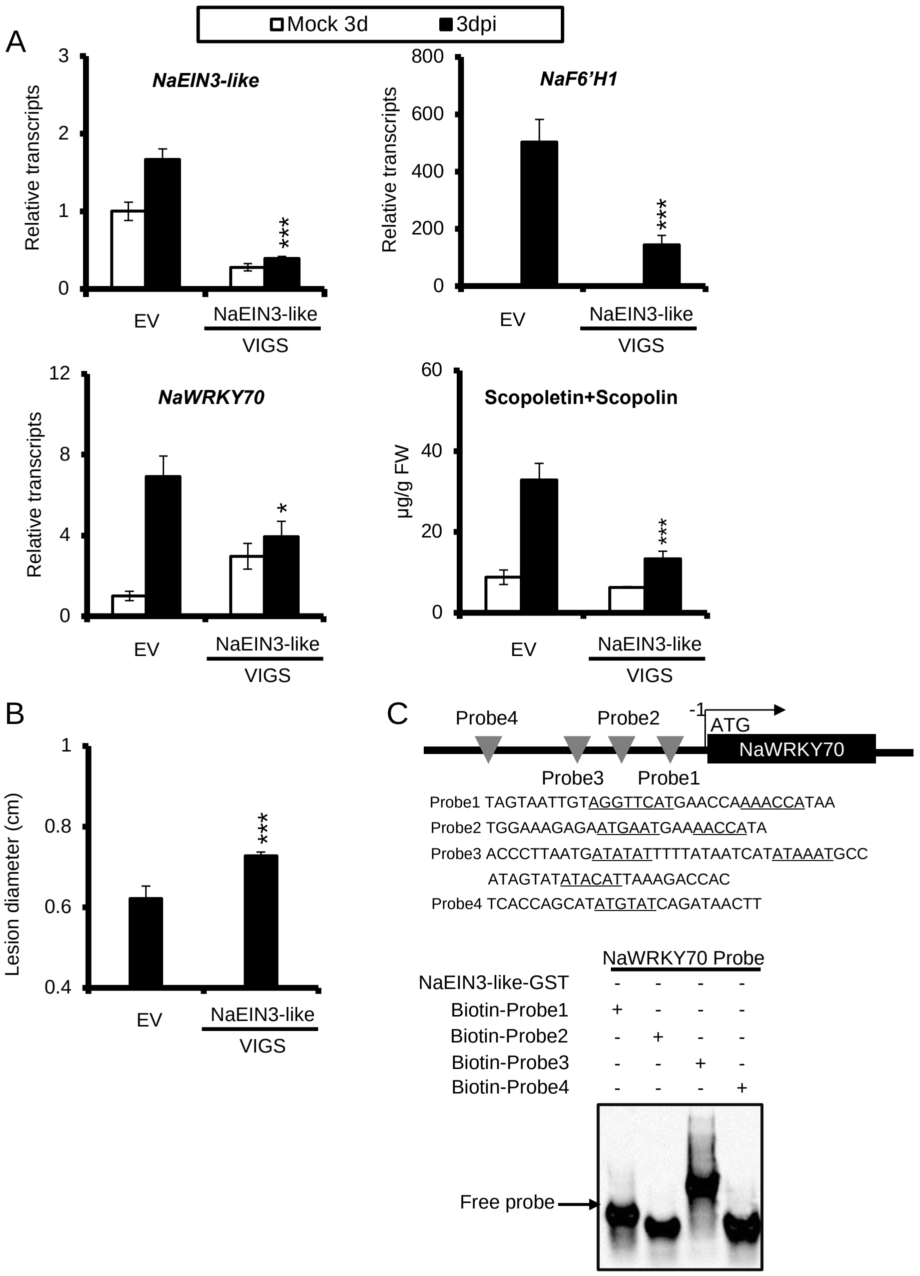

Supplementary Fig. S7. *NaEIN3-like* is required for *A. alternata*-induced transcripts of *NaWRKY70* and *NaF6'H1*, scopoletin and scopolin production, and plant resistance to *A. alternata*. However, NaEIN3-like proteins cannot bind to the probes designed from the *NaWRKY70* promoter.

(A) Mean (±SE) relative expression levels of *NaEIN3-like, NaF6'H1, NaWRKY70* and scopoletin and scopolin levels were measured in five biological replicated young leaves of EV and VIGS NaEIN3-like plants at 3 dpi. Asterisks indicate the level of significant

differences between WT and VIGS NaEIN3-like plants after infection by *A. alternata* (Student's *t*-test: *, P<0.05; ***, P<0.005).

(B) Mean (± SE) diameter of necrotic lesions of 15 biological replicated young leaves of EV and VIGS NaEIN3-like infected with *A. alternata* for 5 d. Asterisks indicate the level of significant differences between WT and VIGS NaEIN3-like plants (Student's *t*-test: ***, P<0.005).

(C) Schematic diagrams of the *NaWRKY70* promoter and candidate probe sequences. EMSA results demonstrated that NaEIN3-like protein could not bind to four predicted probes designed from the *NaWRKY70* promoter.

```
NaMYC2a    1 --------MTDYRIPTMNN-IWSNTTSDDNMMEAFLSSDPSSFWPGTTTTPTPRTSVSPAPPPVTSIAGDPLKSMPYFNQESL  74
NaMYC2b    1 MNLWNTSGTTDDNVSMMEAFMSSDLTSFWATSNSTAAAVTSNSDHIPVNTPTVLLPSS-CASTVTAVAVDASKSMPFFNQETL  82
NaMYC2c    1 ------------------------------------------------------------MEDITSTS----------STNTL  13
NaMYC2d    1 --------MTDYRLPTMNNNIWS------------------------STTPTPRTSVSPALVSVTGTTGDPLKSMAFFSVESL  51

NaMYC2a   75 QQRLQTLIDGAREAWTYAIFWQSSVVDFASPSVLGWGDGYYKGEEDKNKRKTASFSPDFITEQAHRKKVLRELNSLISGTQTG 157
NaMYC2b   83 QQRLQTLIDGARETWTYAIFWQSSVVDLTSPFVLGWGDGYYKGEEDKAGRKLAVSSPAYIAEQEHRKKVLRELNSLISCTQTG 165
NaMYC2c   14 QKHLQYIIHSRQEWWVYAIFWQAS-KDANGRLIFSWGDGHFRGTKDLANSAKVRIP---------------------------  68
NaMYC2d   52 QQRLQTLIDGAREAWTYGIFWQSDVVDFASPLVLRWGNGYYNGEEDKNECKTTSFSRYFIAEQEHRKKVLREVNSLISGTQNG 134

NaMYC2a  158 GENDAVDEEVTDTEWFFLISMTQSFVNGSGLPGLAMYSSSPIWVTGAERLAASHCERARQAQGFGLQTIVCIPSANGVVELGS 240
NaMYC2b  166 -TDDAVDEEVTDTEWFFLISMTQSFVNGSGLPGQALYNSSPIWVAGAEKLAASHCERARQAQGFGLQTMVCIPSANGVVELGS 247
NaMYC2c   69 ----NVDNNVSDTELFYMVSVSKYFVADNELIVRAYNPTSYIWLNNYHELQLYNYDRAKEAHLHGIRTLVCIPTPNGVVELGS 147
NaMYC2d  135 GENDVVDEVVTDTEWFFLISMSESFVNGSGLPGLAMHNSSPIWVTGTERLSASNCERARQAQRYGLQTMFCIPSANGVVELGS 217

NaMYC2a  241 TELIFQTADLMNKVKVLFNFNIDMGATTGSGSGSCAIHAEPDPSALWLTDPASSAVEVKDSNTVPSSNSSKQLVFGNENS--- 320
NaMYC2b  248 TELIIQSSDLMNKVRVLFNFNNDLGS------GSWAVQPESDPSALWLTDPSPAAVQVKDLNTVPSSNSSKQVVFDNENNGHI 324
NaMYC2c  148 SEIIQENWDLVQLSRSLFGLSN----------NNIITPSPINHQGLFSYNFVSLGENHKVENDSPQATDSKLKQETVDGN--- 217
NaMYC2d  218 TELISQSSDLMDKVKVLFNFNTDMSSPTGLVSGSRVVESEPDLSALRLTDP-------------------------------- 268

NaMYC2a  321 ---ENGNQNSQQTQGFFTRELNFSEYGFDGSNTRNGNANSSRSCKPESGEILNFGDSTKRSASSANGSLFSGQSQFGPGSAEE 400
NaMYC2b  325 CDNQQQHHSQQQTQGFFTRELNFSEFGFDGSS-NNRNGNSSVSCKPESGEILNFGDSTKKSAN---GNLFSGQSHFGAGE--- 400
NaMYC2c      -----------------------------------------------------------------------------------
NaMYC2d      -----------------------------------------------------------------------------------

NaMYC2a  401 NKNKNKKRSPASRGSNDEGMLSFVSGVILPSSN----TGKSGGGGDSDQSDLEASVVKEADSSRVVDPEKKPRKRGRKPANGR 479
NaMYC2b  401 -ENKNKKRSPASRGSNEEGMLSFVSGTILPAASGAMKSSGCVGEDSSDHSDLEASVVKEAESSRVVEPEKRPKKRGRKPANGR 482
NaMYC2c  218 -----------------------------------------ISLGNSDSFENESSTINNIN-----RPIKRGRKSSSSNATRT 254
NaMYC2d      -----------------------------------------------------------------------------------

NaMYC2a  480 EEPLNHVEAERQRREKLNQRFYALRAVVPNVSKMDKASLLGDAIAFINELKSKVQNSDSDKEELRNQIESLRKELANKGSNYT 562
NaMYC2b  483 EEPLNHVEAERQRREKLNQRFYALRAVVPNVSKMDKASLLGDAISYINELKLKLQNTETDREDLKSQIEDLKKELASEDSWRP 565
NaMYC2c  255 EMAKNHVEAERQRREKLNNRFYALRSVVPNVSKMDRASLLADAVTYINELKAKVEELESYKILSQKPKRQCATNSVQTVPS-- 335
NaMYC2d      -----------------------------------------------------------------------------------

NaMYC2a  563 GPPP-------SNQDLKILDMDIDVKVIGWDAMIRIQSNKKNHPAARLMAALMELDLDVHHASVSVVNELMIQQATVKMGSRL 638
NaMYC2b  566 GPPPNQDHKMSSHTGSKIVDVDIDVKIIGWDAMVRIQCNKKNHPAARLMVALKELDLEVHHASVSVVNDLMIQQATVKMGSRL 648
NaMYC2c  336 ---------TVVNRANNSFGMEVEVKIIGLEAMVRVRSPDVNYPCARLMNVLRELELQVNHASVSSVKNLMLQDVVIRVPNEV 409
NaMYC2d      -----------------------------------------------------------------------------------

NaMYC2a  639 YTQEQLRISLTSRIAESR                                                                  656
NaMYC2b  649 YTEEQLRIALTSRVAETR                                                                  666
NaMYC2c  410 ANEEVLKSVILKRLSVAN                                                                  427
NaMYC2d      ------------------
```

Supplementary Fig. S8. Protein sequence alignment of NaMYC2a, NaMYC2b, NaMYC2c and NaMYC2d.

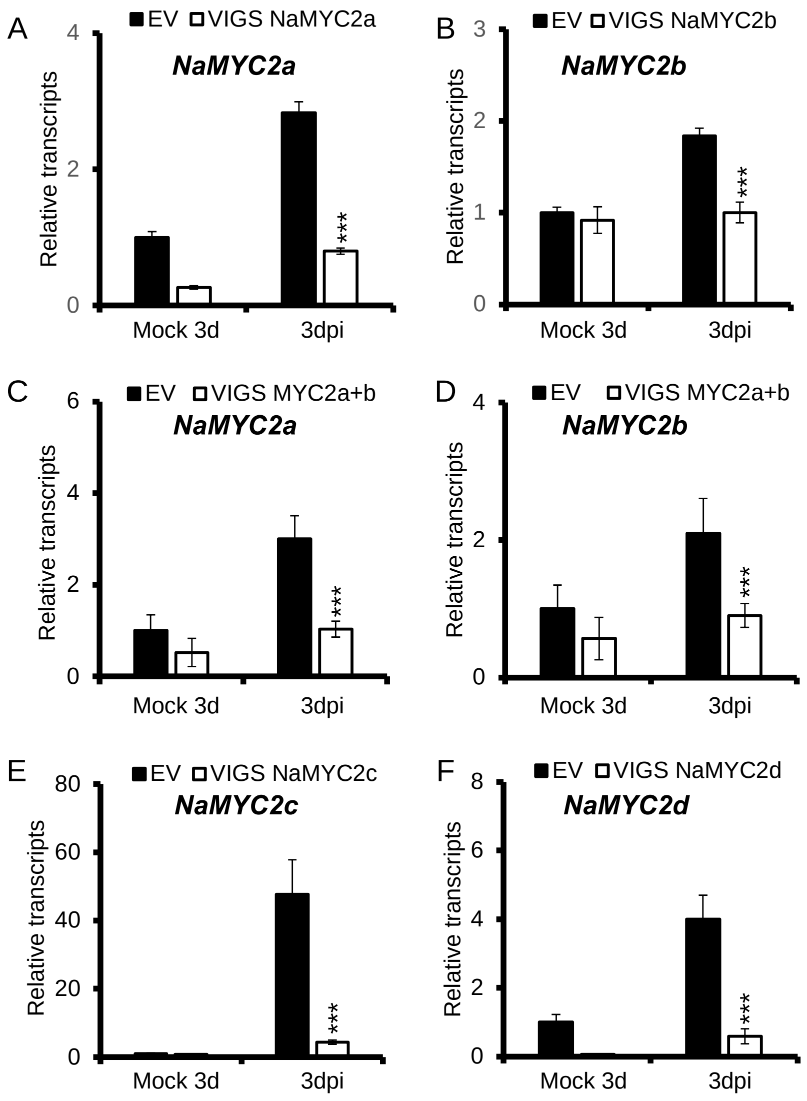

Supplementary Fig. S9. *NaMYC2a, NaMYC2b, NaMYC2c,* and *NaMYC2d* were all successfully silenced by VIGS.

(A) Mean (±SE) relative expression levels of *NaMYC2a* were measured in five biological replicated young leaves of EV and VIGS NaMYC2a plants at 3 dpi. Asterisks indicate the level of significant differences between WT and VIGS NaMYC2a plants after infection by *A. alternata* (Student's *t*-test: ***, P<0.005).

(B) Mean (±SE) relative expression levels of *NaMYC2b* were measured in five biological replicated young leaves of EV and VIGS NaMYC2b plants at 3 dpi. Asterisks indicate the level of significant differences between WT and VIGS NaMYC2b plants after infection by *A. alternata* (Student's *t*-test: ***, P<0.005).

(C) Mean (±SE) relative expression levels of *NaMYC2a* were measured in five biological replicated young leaves of EV and VIGS NaMYC2a+b plants at 3 dpi. Asterisks indicate the level of significant differences between WT and VIGS NaMYC2a+b plants after infection by *A. alternata* (Student's *t*-test: ***, $P<0.005$).

(D) Mean (±SE) relative expression levels of *NaMYC2b* were measured in five biological replicated young leaves of EV and VIGS NaMYC2a+b plants at 3 dpi. Asterisks indicate the level of significant differences between WT and VIGS NaMYC2a+b plants after infection by *A. alternata* (Student's *t*-test: ***, $P<0.005$).

(E) Mean (±SE) relative expression levels of *NaMYC2c* were measured in five biological replicated young leaves of EV and VIGS NaMYC2c plants at 3 dpi. Asterisks indicate the level of significant differences between WT and VIGS NaMYC2c plants after infection by *A. alternata* (Student's *t*-test: ***, $P<0.005$).

(F) Mean (±SE) relative expression levels of *NaMYC2d* were measured in five biological replicated young leaves of EV and VIGS NaMYC2d plants at 3 dpi. Asterisks indicate the level of significant differences between WT and VIGS NaMYC2d plants after infection by *A. alternata* (Student's *t*-test: ***, $P<0.005$).

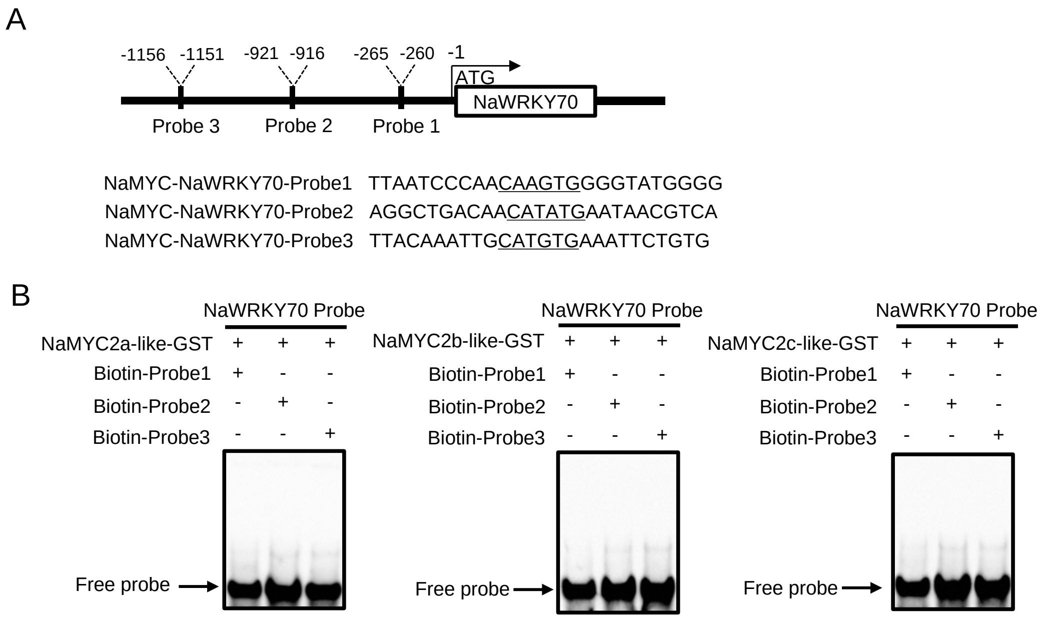

Supplementary Fig. S10. NaMYC2a, NaMYC2b and NaMYC2c cannot bind to the three probes designed from the *NaWRKY70* promoter.

(A) Schematic diagrams of the *NaWRKY70* promoter and probe sequences.

(B) EMSA results demonstrated that NaMYC2a, MaMYC2b or NaMYC2c protein could not bind to three predicted probes designed from the *NaWRKY70* promoter.
